# Rates of ultrasonic vocalizations are more strongly related than acoustic features to non-vocal behaviors in mouse pups

**DOI:** 10.3389/fnbeh.2022.1015484

**Published:** 2022-12-19

**Authors:** Nicole M. Pranic, Caroline Kornbrek, Chen Yang, Thomas A. Cleland, Katherine A. Tschida

**Affiliations:** Department of Psychology, Cornell University, Ithaca, NY, United States

**Keywords:** ultrasonic, vocalization, mouse, pup, isolation

## Abstract

Mouse pups produce. ultrasonic vocalizations (USVs) in response to isolation from the nest (i.e., isolation USVs). Rates and acoustic features of isolation USVs change dramatically over the first two weeks of life, and there is also substantial variability in the rates and acoustic features of isolation USVs at a given postnatal age. The factors that contribute to within age variability in isolation USVs remain largely unknown. Here, we explore the extent to which non-vocal behaviors of mouse pups relate to the within age variability in rates and acoustic features of their USVs. We recorded non-vocal behaviors of isolated C57BL/6J mouse pups at four postnatal ages (postnatal days 5, 10, 15, and 20), measured rates of isolation USV production, and applied a combination of pre-defined acoustic feature measurements and an unsupervised machine learning-based vocal analysis method to examine USV acoustic features. When we considered different categories of non-vocal behavior, our analyses revealed that mice in all postnatal age groups produce higher rates of isolation USVs during active non-vocal behaviors than when lying still. Moreover, rates of isolation USVs are correlated with the intensity (i.e., magnitude) of non-vocal body and limb movements within a given trial. In contrast, USVs produced during different categories of non-vocal behaviors and during different intensities of non-vocal movement do not differ substantially in their acoustic features. Our findings suggest that levels of behavioral arousal contribute to within age variability in rates, but not acoustic features, of mouse isolation USVs

## Introduction

Following birth, mouse pups are blind, deaf, and possess limited sensorimotor capacity, relying on their mother for food and thermoregulation (Theiler, 1972; Brust et al., 2015). During their first two weeks of life, they produce vocalizations in the ultrasonic range (i.e., USVs) which are emitted in response to isolation from the dam and cold exposure (Zippelius and Schleidt, 1956; Okon, 1970a; Hahn and Schanz, 2002). These so-called isolation USVs induce a maternal retrieval response and are crucial for the survival of these altricial pups during their first two weeks of life (Noirot, 1972; Ehret and Haack, 1984; Ehret, 1992).

Both rates and acoustic features of mouse isolation USVs change over early development (Grimsley et al., 2011; Rieger and Dougherty, 2016). Rates of isolation USVs of C57 mouse pups start out low shortly after birth, peak at around postnatal day (P) 6–7, and gradually decline as pups gain the ability to thermoregulate and locomote efficiently, disappearing after 2 weeks of age (Okon, 1970a; Hahn et al., 1998; Thornton et al., 2005). With regard to developmental changes in USV acoustic features, previous studies have reported that USV duration increases over early development and then gradually declines as pups approach 2 weeks of age (Liu et al., 2003; Grimsley et al., 2011; Yin et al., 2016; Castellucci et al., 2018). Meanwhile, the mean pitch of isolation USVs becomes less variable over early development (Liu et al., 2003; Grimsley et al., 2011), and the interval between consecutive USVs decreases (Liu et al., 2003; Grimsley et al., 2011; Yin et al., 2016; Castellucci et al., 2018). These developmental changes in the acoustic features of isolation USVs are thought to be driven by physiological changes that take place during the first two postnatal weeks, including changes to the larynx, lung capacity, and vocal-respiratory coordination (Elwood and Keeling, 1982; Dutschmann et al., 2014; Riede et al., 2020; Wei et al., 2021).

Rates and acoustic features of isolation USVs also exhibit substantial variability within a given postnatal age, both between pups and between recordings from the same pup, even when pup and chamber temperature are monitored and maintained in a narrow range (Grimsley et al., 2011; Rieger and Dougherty, 2016; Barnes et al., 2017). Previous studies have explored how olfactory cues (D’Amato and Cabib, 1987; Santucci et al., 1994; Branchi et al., 1998; Olejniczak et al., 1999; Szentgyörgyi and Kapusta, 2004; Ehret, 2005) and tactile cues (Okon, 1970b; Branchi et al., 1998) influence rates of isolation USV production in rodent pups. The factors that contribute to the substantial variability in rates and acoustic features of isolation USVs that occurs even in the absence of these specific environmental and social cues, however, remain unknown.

One factor that may contribute to within age variability in the rates and acoustic features of isolation USVs are the non-vocal behaviors exhibited by mouse pups (Fox, 1965). Throughout early development, motor abilities such as locomotion and grooming develop rapidly and become increasingly refined throughout the postnatal period (Fox, 1965; Brust et al., 2015). Different non-vocal behaviors might differentially influence laryngeal and vocal tract configuration and are likely associated with different rates of respiration and levels of behavioral arousal, factors which may in turn influence rates and acoustic features of USVs. To date, only a single study has examined the relationship between non-vocal behavior and variability in the rates and acoustic features of isolation USVs (Branchi et al., 2004). The authors report that P7 CD-1 Swiss mouse pups produce higher rates of isolation USVs when they engage in locomotion relative to other non-vocal behaviors (Branchi et al., 2004). However, the extent to which non-vocal pup behaviors relate to within age variability in rates and acoustic features of isolation USVs, and how these relationships change over early development, remains poorly understood.

To address these questions, we recorded isolation USVs and non-vocal behaviors of male and female C57BL/6J mice at four postnatal ages (P5, P10, P15, and P20). At each age, we quantified rates of isolation USVs and examined USV acoustic features, using pre-defined features as well as an unsupervised machine learning-based acoustic analysis method. Categories of non-vocal behaviors were scored by trained observers, and we also quantified movement intensity from videos using an annotation and instance segmentation-based animal tracking and behavior analysis package. We then compared these descriptions of vocal and non-vocal behavior to test the hypothesis that pup non-vocal behaviors are related to within age variability in the rates and acoustic features of isolation USVs.

## Materials and methods

Further information and requests for resources should be directed to the corresponding author, KT (kat227@cornell.edu).

### Subjects

Male (*N* = 21) and female (*N* = 24) C57BL/6J mice (Jackson Laboratories, 000664) were housed with their siblings and both of their parents until weaning at postnatal day 21. Mice were kept on a 12 h:12 h reversed light/dark cycle and given *ad libitum* food and water for the duration of the experiment. Because we inadvertently failed to save *N* = 2 audio recordings from P5 mice and *N* = 1 audio recording from P10 mice, the sample sizes for audio recordings within each age group are as follows: P5, *N* = 43 mice; P10, *N* = 44 mice; P15, *N* = 45 mice; P20, *N* = 45 mice. Because we inadvertently failed to save *N* = 3 video recordings from P5 mice, *N* = 1 video recording from P10 mice, and *N* = 1 video recording from P15 mice, the sample sizes for video recordings within each age group are as follows: P5, *N* = 42 mice; P10, *N* = 44 mice; P15, *N* = 44 mice; P20, *N* = 45 mice. For the comparison of USV rates and features to non-vocal behavior categories, the sample sizes within each age group are as follows: P5, *N* = 42 mice; P10, *N* = 44 mice; P15, *N* = 44 mice.

### Study design

Measurements of the vocal and non-vocal behaviors of young mice were recorded longitudinally across early development, beginning at postnatal day 5 and repeated every fifth day until postnatal day 20. Pups were identified and tracked individually by placing markings on their tails with permanent markers that were renewed every other day. For USV recordings, pups were placed in a custom acrylic chamber inside a sound-attenuating recording chamber (Med Associates) equipped with an ultrasonic microphone (Avisoft), infrared light source (Tendelux), and webcam (Logitech, with the infrared filter removed to enable video recording under infrared lighting). To elicit the production of isolation USVs, pups were recorded alone in the chamber for 5 min (isolation sessions). As part of a different experiment aimed at testing the effects of social partners on the production of isolation-induced USVs, pups were then exposed to either their mother (dam social group), a novel adult female (novel female social group), or no social partner (control group) for 5 min (social sessions). In the final 5 min, pups were again recorded alone in the chamber (re-isolation sessions). USV data from isolation and re-isolation sessions were pooled for each mouse in the current study, as we found that re-isolation USV rates did not differ between groups, nor did the acoustic features of USVs produced during isolation and re-isolation sessions differ (Supplementary Figures 1A–C). Ambient temperature inside the test chamber was maintained between 21.5 and 23°C (min/max recorded across all trials; mean chamber temperature was 22.38°C ± 0.32, *N* = 138 trials). To minimize temperature drops during transfer into the test chamber, pups were brought into the testing room within their home cage and were placed into the test chamber immediately before the start of the trial. To assess the consistency of pup body temperature changes within each age group, we also used a thermal camera to measure pup temperature in a small number of trials. At the beginning of the trial, the mean and standard deviation of body temperature was 33.89°C ± 0.19 for P5 pups (*N* = 6), 33.64°C ± 0.37 for P10 pups (*N* = 6), and 32.77°C ± 0.52 for P15 pups (*N* = 5). Over the course of the trial, body temperature declined 4.97°C ± 0.37 in P5 pups (*N* = 6), 0.51°C ± 0.27 in P10 pups (*N* = 6), and increased on average 1.05°C ± 0.72 in P15 pups (*N* = 5).

### USV recording, detection, and calculation of pre-defined acoustic features

USVs were recorded using an ultrasonic microphone (Avisoft, CMPA/CM16), connected to an Avisoft recording system (UltrasoundGate 116H, 250 kHz sample rate). USVs were detected using custom MATLAB codes (Tschida et al., 2019) with the following parameters implemented to detect USVs: mean frequency > 45 kHz; spectral purity > 0.3; spectral discontinuity < 1.00; minimum USV duration = 5 ms; minimum inter-syllable interval = 30 ms). For every detected USV syllable, we calculated eight acoustic features: (1) duration (ms), (2) inter-syllable interval (ISI, defined as the interval from the onset of one USV to the onset of the next USV, > 400 ms intervals excluded from analyses), (3) mean pitch (measured in kHz; dominant frequency calculated at each time point of the USV, then averaged across entire syllable), (4) pitch slope (calculated as difference in dominant frequency from start to end of a USV divided by USV duration), (5) mean frequency (calculated after thresholding spectrograms to eliminate frequencies < 25 kHz and > 110 kHz; Holy and Guo, 2005), (6) standard deviation of mean frequency, (7) spectral purity (fraction of USV power concentrated into a single frequency bin; Holy and Guo, 2005), and (8) frequency modulation index (FM index). FM index was calculated by normalizing the duration of each USV [time points were divided by USV duration to range from 0 (onset) to 1 (offset)]. The dominant frequency of the USV over time was then divided by the mean pitch, converted to a log scale, and the standard deviation of these normalized frequency values was defined as the FM index (Saito et al., 2019).

### Analyzing USV acoustic features with autoencoded vocal analysis

Isolation USVs detected with custom MATLAB codes were analyzed using Autoencoded Vocal Analysis (AVA) v0.2 (Goffinet et al., 2021), a Python package to describe and quantify animal vocalizations. Briefly, AVA is a data-driven approach to acoustic analysis that does not explicitly consider any pre-defined acoustic features, but rather, learns directly from data (spectrograms of USV syllables; i.e., high-dimensional representations of audio data) to project USVs into low dimensional latent feature spaces. To do this, AVA uses a variational autoencoder (VAE; Kingma and Welling, 2014), an unsupervised learning method that learns from the data by training two probabilistic maps, an encoder and a decoder. Both maps are parameterized *via* deep convolutional neural networks. Spectrograms of individual USVs are encoded into latent representations and decoded to create reconstructions. We used the following pre-processing parameters to train the VAE: min_freq = 30 kHz, max_freq = 110 kHz, nperseg = 1,024, noverlap = 512, spec_min_val = 3.3, spec_max_val = 7.0, mel = False, time_stretch = True, within_syll_normalize = False. The multi-dimensional latent space was visualized by compressing the output of the VAE into 2-dimensional space using a uniform manifold approximation and projection (UMAP) algorithm (McInnes et al., 2020).

We quantified differences between distributions of latent syllable representations with Maximum Mean Discrepancy (MMD) (Gretton et al., 2012), based on the procedure described by Goffinet et al. (2021). MMD is a non-parametric difference measure between pairs of distributions. MMD represents the difference between two probability distributions based on the mean embeddings of these distributions in a reproducing kernel Hilbert space. To test whether USVs produced during isolation and re-isolation sessions differed in their acoustic features, we established a baseline comparison for each age group by assigning pups to one of two groups and then calculating MMD between these two distributions of isolation USVs (within session comparisons, Supplementary Figures 1B, C). We then compared these MMD values to MMD values calculated between distributions of latent syllable representations from isolation vs. re-isolation sessions within each age group (between sessions comparisons, Supplementary Figure 1). To determine whether pups from different age groups produced USVs with different acoustic features, we first established the baseline level of acoustic variability within each age by considering USVs produced during isolation sessions vs. USVs produced during re-isolation sessions and calculating MMD between these two distributions (Supplementary Figure 1). We then compared these within age MMD values to MMD values quantifying the difference between distributions of latent syllable representations from pairs of different ages (P5 vs. P10, P5 vs. P15, and P10 vs. P15; Supplementary Figure 1). Finally, to compare the acoustic features of USVs produced in each age group during different non-vocal behaviors (see next section), MMD values were calculated between distributions of latent syllable representations produced during a single category of non-vocal behavior (within behavior comparisons, pups from each age split into two groups as above) or produced during different categories of non-vocal behaviors (between behavior comparisons).

### Analysis of categories of non-vocal behaviors

To examine how categories of non-vocal behaviors of isolated mice changed from P5 to P15, trained observers scored the following behaviors from webcam videos during isolation and re-isolation sessions: (1) locomotion [movement of forelimbs and/or head while four limbs are touching the surface of the testing chamber; both locomotion (P15) and attempted locomotion (P5 and P10 mice) were scored]; (2) wriggling (mouse on its side or in supine position, accompanied by movement of forelimbs and/or head); (3) lying still (no visible movement); and (4) grooming (see also Supplementary Movies 1–4). Behavior scores of two trained observers were averaged (inter-rater reliability: *r* = 0.99) and used in analyses.

### Analysis of non-vocal movement intensity and comparison to USV rates

To enable comparisons of USV rates to the intensity of non-vocal movements, a Python software package for behavior analysis, Annolid,^1^ was used to quantify non-vocal movement intensity in each trial. Briefly, the mouse pup was detected and segmented in each video frame with an instance segmentation model trained on a custom labeled dataset, generating an instance mask of the pup’s entire body shape surrounding its position in each frame. The optical flow across consecutive pairs of frames was then calculated and filtered with the pup mask to generate a vector of movement intensity derived from the motion of this whole-body mask, which includes head, body, and limb movements. The movement vector then was smoothed with a moving average filter (span = 90 frames) and linearly interpolated such that the resulting movement vector was on the same time base as the USV rate vector for each trial. USV rates were calculated for each trial by counting the number of USVs produced in 3s-long bins. To compare USV rates to movement intensity within individual trials, the cross-covariance between movement intensity and USV rate was calculated for each trial using the xcov function in Matlab with the “coeff” scaling option, which normalizes each vector such that the auto-covariance at time lag 0 equals 1. Thus, each cross-covariance has a maximum possible value of 1 and a minimum possible value of −1, allowing us to pool data from different trials to compare the strength of the cross-covariance between age groups. Matched comparisons were generated for each trial by comparing movement and USVs from the first 5 min of recording (isolation movement vs. isolation USVs) and the final 5 min of recording (re-isolation movement vs. re-isolation USVs). Shuffled comparisons were generated for each trial by comparing movement and USVs from non-corresponding 5-min recording periods (isolation movement vs. re-isolation USVs, re-isolation movement vs. isolation USVs). These matched and shuffled comparisons were averaged to generate a mean matched and mean shuffled cross-covariance for each trial. A subset of trials in which 0 USVs were recorded were excluded from these analyses (*N* = 2 P5 trials and *N* = 19 P15 trials), as cross-covariances could not be calculated for these trials. Matched and shuffled cross-covariances from multiple pups from the same age group were averaged together to obtain a mean pooled matched and a mean pooled shuffled cross-covariance for each age group. Mean pooled covariance coefficients (CCs) were calculated as the maximum value of the mean pooled matched and mean pooled shuffled cross-covariances between time lags of −5 and +5 s for each age group. The CC for each individual trial was then calculated by measuring the value of the single trial cross-covariance at the time lag of the mean pooled CC. To calculate the significance of the cross-covariance between movement intensity and USV rates, CCs were compared for matched comparisons and for shuffled comparisons within each age group.

### Statistical analyses

To determine whether to use parametric or non-parametric statistical tests for a given comparison, we examined the normality of the residuals for the relevant data distributions (determined by visual inspection of plots of z-scored residuals; cases in which residuals diverged notably from the 45-degree line of a normal distribution were deemed non-normally distributed). Non-parametric tests were applied for analyses of non-normally distributed distributions. For both parametric and non-parametric comparisons, two-sided statistical comparisons were used (alpha = 0.05). All *p* values for pairwise comparisons represent Bonferroni-corrected values. Details of the statistical analyses used in this study are included in Supplementary Table 1. No statistical methods were used to pre-determine sample size. All statistical analyses were carried out using R 4.1.0 (R Core Team, 2021) and R Studio 1.4.1717 (RStudio Team, 2022).

## Results

### Rates and acoustic features of isolation USVs change over early postnatal development

**FIGURE 1:**
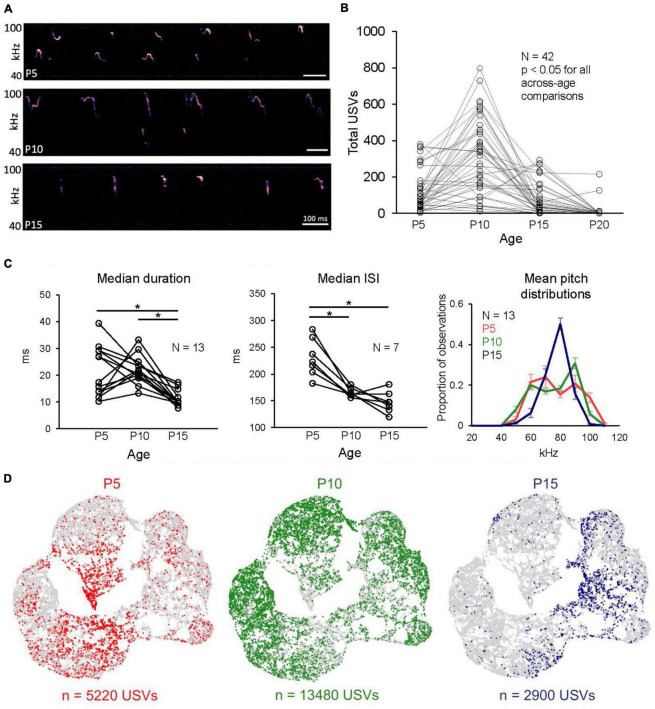
Rates and acoustic features of isolation USVs change over early postnatal development. **(A)** Spectrograms of representative isolation USVs produced by P5, P10, and P15 mice. **(B)** The number of isolation USVs is shown for *N* = 42 pups recorded at P5, P10, P15, and P20. **(C)** (Left) Median values of USV duration vs. age are plotted for *N* = 13 mice that produced > 50 USVs at P5, P10, and P15 (*p* < 0.01 for P5 vs. P15 and P10 vs. P15, Friedman test followed by *post-hoc* tests). (Middle) Same, for median ISIs (*p* < 0.01 for P5 vs. P10, *p* = 0.02 for P5 vs. P15, Friedman test followed by *post-hoc* tests). Note that because ISIs > 400 ms are excluded, there are only *N* = 7 mice with > 50 ISIs at each age. (Right) Distributions of mean pitch are plotted from the same *N* = 13 pups. Error bars in right panel are S.E.M. **(D)** UMAP projections of latent syllable representations of USVs produced by P5 (red), P10 (green), and P15 mice (blue). Points represent individual syllables and are closer to each other if acoustically more similar.

We first measured how rates of USV production changed over early postnatal development by tracking and comparing USV rates from mouse pups recorded for 10 min in isolation at P5, P10, P15, and P20 (Figures 1A, B; see Methods). We found that rates of isolation USVs increased from P5 to P10, and that P10 mice vocalized at significantly higher rates than all other ages (Figure 1B; P5: 120.5 ± 109.7 USVs; P10: 312.0 ± 205.0 USVs; P15: 68.8 ± 88.5 USVs; P20: 8.9 ± 38.1; *N* = 42 mice, *p* < 0.05 for P5 vs. P15, and *p* < 0.001 for all other comparisons; one-way ANOVA with repeated measures followed by *post-hoc* tests). We conclude that the production of isolation USVs by mouse pups peaks around P10 and then gradually declines, consistent with previous studies (Liu et al., 2003; Grimsley et al., 2011; Yin et al., 2016; Castellucci et al., 2018).

Next, we confirmed that we were able to detect developmental changes in the acoustic features of isolation USVs produced by P5, P10, and P15 mice, similar to those reported in previous studies (P20 mice were not considered further in the study, as they produced near-zero rates of isolation USVs, Figure 1B and Supplementary Table 2; only 2 of 45 P20 pups produced > 25 USVs). Previous studies have described acoustic changes in isolation USVs over early development by quantifying pre-defined acoustic features or by examining the proportions of USVs that fall into experimenter-defined acoustic categories (Liu et al., 2003; Scattoni et al., 2008; Grimsley et al., 2011; Yin et al., 2016; Castellucci et al., 2018; Vogel et al., 2019; Fonseca et al., 2021). To assess whether the USVs recorded in our dataset exhibit similar developmental changes to those that have been previously reported, we first quantified developmental changes in USVs using a small number of pre-defined acoustic features (see Methods; see Supplementary Figure 2 for analyses of additional pre-defined features). Because some mice within each age group produced low rates of isolation USVs (Figure 1B), we analyzed changes in acoustic features across development only from mice that produced moderate rates of USVs at each time point considered (*N* = 13 mice produced > 50 USVs at P5, P10, and P15; see also Supplementary Table 2). We found that USV durations decreased in P15 mice relative to earlier ages and that the interval between consecutive USV syllables (i.e., ISI) was highest at P5 and decreased at later ages (Figure 1C, left and middle panels; mean median and standard deviation of duration for P5 USVs: 21.35 ± 9.41; P10 USVs: 22.06 ± 5.29; P15 USVs: 11.30 ± 3.00; *p* < 0.01 for P5 vs. P15 and for P10 vs. P15; mean median and standard deviation of ISIs for P5 USVs: 230.73 ± 35.53; P10 USVs: 165.63 ± 8.38; P15 USVs: 147.39 ± 19.89; *p* < 0.01 for P5 vs. P10 and *p* = 0.02 for P5 vs. P15; Friedman tests followed by *post-hoc* tests). In addition, distributions of mean pitch changed dramatically in shape from bimodal at P5 and P10 to unimodal at P15, and mean pitch was also less variable at P15 than at earlier ages (Figure 1C, right; mean standard deviation of mean pitch for P5 USVs: 12.97 ± 2.72 kHz; for P10 USVs: 13.68 ± 1.12 kHz; for P15 USVs: 7.87 ± 1.68 kHz; *p* < 0.01 for P5 vs. P15 and for P10 vs. P15; Friedman test followed by *post-hoc* tests). These changes in rates and acoustic features of USVs are consistent with those reported in previous studies (Liu et al., 2003; Grimsley et al., 2011; Yin et al., 2016; Castellucci et al., 2018).

A potential limitation of examining USVs using pre-defined acoustic features or syllable categories is that pre-defined features may miss important aspects of acoustic variability and may be correlated with one another, and USVs that fall within a given experimenter-defined acoustic category still exhibit substantial variability in their acoustic features (Sainburg et al., 2020; Goffinet et al., 2021). To examine developmental changes in the acoustic features of isolation USVs in a manner that does not depend on pre-defined acoustic features or clustering of USVs into categories, we examined and compared vocal repertoires of mice using Autoencoded Vocal Analysis v0.2 (AVA; Goffinet et al., 2021), a recently described unsupervised modeling approach that uses VAEs (Kingma and Welling, 2014). In this method, VAEs use spectrograms of individual vocalizations as inputs, apply an encoder to represent and compress these spectrograms into a small number of latent features, and then use a decoder to reconstruct the input spectrograms (see Supplementary Figure 3 for reconstructed spectrograms of representative syllables). We provided spectrograms of isolation USV syllables produced by P5-P15 mice as input to train the model and found that the VAE converged on a latent representation of four dimensions. We then compressed the resulting latent features with a dimensionality reduction method, uniform manifold approximation and projection (UMAP; McInnes et al., 2020) and visualized latent syllable representations in 2D space with Bokeh plots. These plots revealed that USVs of P5 and P15 mice were distributed in a largely non-overlapping manner, while P10 USVs were distributed relatively evenly across the acoustic space (Figure 1D; see also Supplementary Figure 3 for UMAP representations of USV syllables color-coded by pre-defined acoustic features).

To quantify differences between these distributions, we calculated the Maximum Mean Discrepancy (MMD) between distributions of latent syllable representations to generate three comparisons: P5 vs. P10 USVs, P5 vs. P15 USVs, and P10 vs. P15 USVs. In addition, the baseline level of acoustic variability within each age was estimated by calculating MMDs between USVs recorded during the first 5 min to those recorded during the final 5 min of isolation within each age group (Supplementary Figure 1). These comparisons revealed clear differences in vocal repertoires between P5, P10, and P15 mice, in agreement with our analyses of pre-defined features, and provide us with a rich and comprehensive description of acoustic changes across early development that we used subsequently to interrogate the relationship between USV acoustic features and non-vocal behaviors.

### Non-vocal behaviors of mouse pups change during early development

**FIGURE 2:**
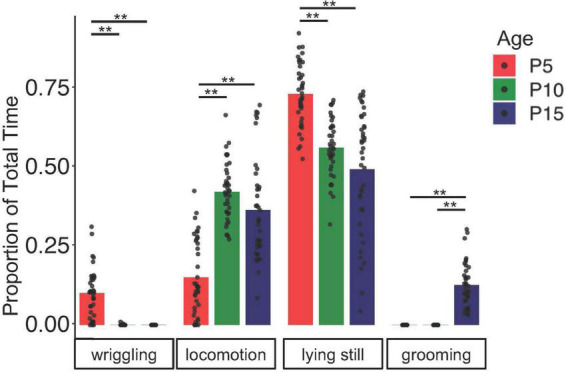
Non-vocal behaviors change over early postnatal development. The proportion of time spent engaged in different non-vocal behaviors is shown for *N* = 40 mice at P5 (red), P10 (green), and P15 (blue). Double asterisks, *p* < 0.01.

To begin to understand how USV rates and acoustic features relate to non-vocal behaviors, we examined how non-vocal behaviors of isolated mice changed from P5 to P15. We recorded four different behaviors produced by mouse pups: grooming, wriggling (movement of forelimbs and/or head while pup is on its side or in supine position), locomotion/attempted locomotion, and lying still (see Methods and Supplementary Movies 1–4). Unsurprisingly, we found that the rates at which mouse pups produce different non-vocal behaviors change over early development (Figure 2; *N* = 40 mice, two-way ANOVA with repeated measures on both factors, followed by *post-hoc* tests). Specifically, we found that P5 mice spend more time lying still and less time locomoting than P10 and P15 mice (proportion time spent at P5 lying still: 0.74 ± 0.10; P10 lying still: 0.57 ± 0.09; P15 lying still: 0.50 ± 0.20; P5 locomotion: 0.15 ± 0.12; P10 locomotion: 0.43 ± 0.09; P15 locomotion: 0.37 ± 0.16; *p* < 0.001 for all comparisons). Wriggling occurred most frequently in P5 mice, declined in frequency in P10 mice, and was no longer observed in P15 animals (proportion time spent wriggling at P5: 0.10 ± 0.08; P10 wriggling: 0.0004 ± 0.002; *p* < 0.001 for P5 vs. P10 and P15 wriggling). Grooming was observed only in P15 mice (proportion time spent grooming at P15: 0.13 ± 0.07; *p* < 0.001 for P15 vs. P5 and P10 grooming). In summary, time spent locomoting increases and time spent lying still declines over early development, wriggling was observed mainly in P5 mice, and grooming was observed only in P15 mice.

### Relationship of USV rates to categories of non-vocal behavior

**FIGURE 3:**
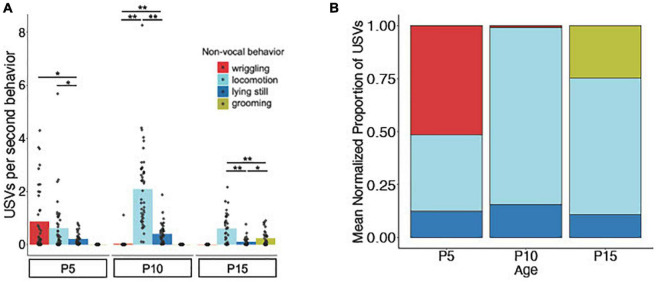
Relationship of USV rates to non-vocal behaviors. **(A)** The mean number of isolation USVs produced per second engaged in four non-vocal behaviors (wriggling, locomotion, lying still, and grooming; see Methods) is shown for P5 (*N* = 42), P10 (*N* = 44), and P15 (*N* = 44) mice. **(B)** Stacked bar plot showing the mean proportion of total USVs produced during each non-vocal behavior normalized by the amount of time each mouse spent performing that behavior. Single asterisk, *p* < 0.05; double asterisks, *p* < 0.01.

We next investigated the relationship between USV rates and categories of non-vocal behaviors of isolated mice. In other words, are mouse pups more likely to produce USVs during certain non-vocal behaviors compared to others, and do these relationships change with age? First, we assigned a behavior code to each USV syllable that corresponds to the non-vocal behavior the mouse was performing when producing that syllable (locomotion, lying still, wriggling, or grooming; see Supplementary Figure 4 for ethograms of USV rates aligned with categories of non-vocal behavior for representative trials). Because there is substantial variability both within ages and across ages in the proportion of time that mice spend engaged in different non-vocal behaviors (Figure 2), we then calculated the numbers of USVs produced by each mouse during different non-vocal behaviors as a function of the total time spent that mouse engaged in each non-vocal behavior (USVs per second of behavior; Figure 3A). This analysis revealed that P5 mice produced the highest normalized rates of USVs during wriggling, followed by locomotion (mean USVs/second at P5 for locomotion: 0.61 ± 1.01; lying still: 0.21 ± 0.22; wriggling: 0.87 ± 1.20; *p* < 0.05 for the comparison between locomotion vs. lying still and lying still vs. wriggling; Friedman test followed by *post-hoc* tests). Both P10 mice and P15 mice produced the highest normalized rates of USVs during locomotion (mean USVs/second at P10 for locomotion: 2.09 ± 1.49; lying still: 0.39 ± 0.37; wriggling: 0.03 ± 0.17; *p* < 0.001 for all comparisons at P10; mean USVs/second at P15 for locomotion: 0.60 ± 0.56; lying still: 0.10 ± 0.15; grooming: 0.23 ± 0.28; *p* < 0.001 for comparisons between locomotion vs. grooming and locomotion vs. lying still at P15; Friedman test followed by *post-hoc* tests). P15 mice also produced higher normalized rates of USVs during grooming than during lying still (mean USVs/second for grooming: 0.23 ± 0.28; lying still: 0.10 ± 0.15; *p* < 0.05; Friedman test followed by *post-hoc* tests). We conclude that, when considering the amount of time mice spent engaged in different non-vocal behaviors, mice in all age groups produce the highest rates of USVs during active behaviors (locomotion, wriggling, and grooming) and are less likely to vocalize while lying still (Figure 3B).

### Relationship of USV acoustic features to categories of non-vocal behavior

**FIGURE 4:**
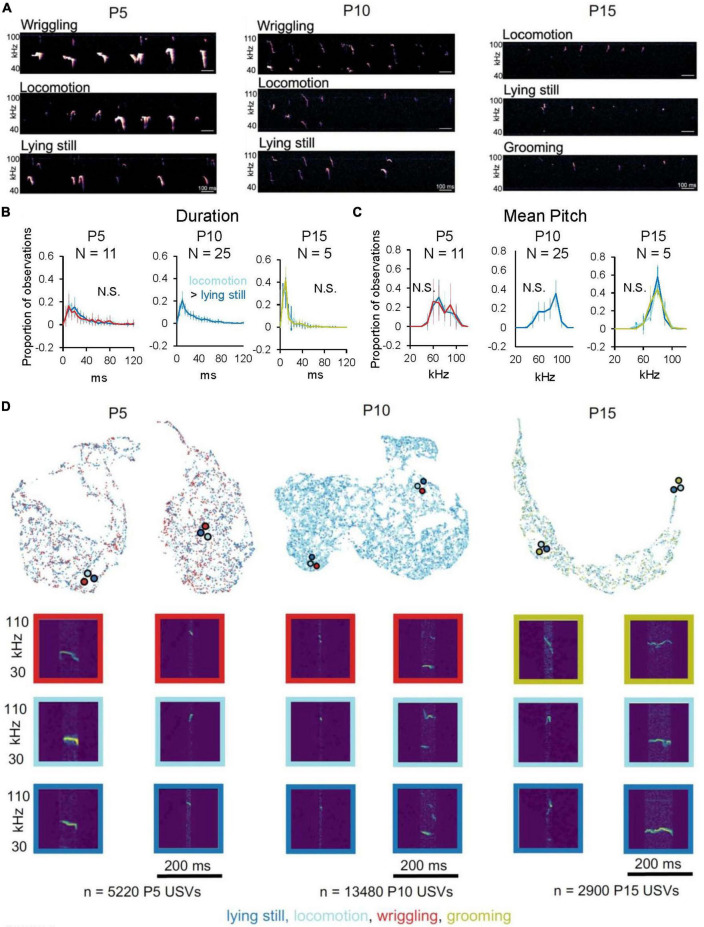
Relationship of USV acoustic features to non-vocal behaviors. **(A)** Spectrograms of representative isolation USVs produced by P5 (left), P10 (middle), and P15 mice (right) during different non-vocal behaviors. **(B)** The mean distributions of durations of isolation USVs produced during different non-vocal behaviors are shown for P5 (left, *N* = 11), P10 (middle, *N* = 25), and P15 mice (right, *N* = 5). P10 mice produced < 50 total USVs during wriggling, so P10 wriggling USVs are excluded from analysis. **(C)** The mean distributions of mean pitch of isolation USVs produced during different non-vocal behaviors are shown for P5 (left, *N* = 11), P10 (middle, *N* = 25), and P15 mice (right, *N* = 5). Significant differences in median values of USV acoustic features between different non-vocal behaviors are indicated by text in each panel; *p* < 0.05 for indicated comparisons. **(D)** UMAP projections of latent descriptions of USV syllables produced by P5 (left), P10 (middle), and P15 mice (right). Representations of individual syllables are outlined in color according to the non-vocal behavior that occurred while that syllable was produced: lying still (dark blue), locomotion (light blue), wriggling (red), and grooming (chartreuse). Example spectrograms are depicted below, and color-coded dots with black outlines indicate landmarks on the Bokeh plot for the representative spectrograms. Number of USVs at P5 produced during wriggling: 1,409, lying still: 2,017, locomotion: 1,489; P10 USVs produced during wriggling: 41, locomotion: 10,410, lying still: 2,912; P15 USVs produced during locomotion: 1,915, lying still: 478, grooming: 510.

We next asked whether mouse pups produce isolation USVs with different acoustic features as they engage in different categories of non-vocal behaviors. We first examined whether USVs produced during different non-vocal behaviors differ in terms of pre-defined acoustic features, considering only mice that produced > 10 USVs during each non-vocal behavior in a given age group (*N* = 11 P5, *N* = 25 P10, and *N* = 5 P15 mice; Figures 4A–C; see Supplementary Figures 5, 6 for analyses of additional pre-defined features and for plots of acoustic feature median values for each age and mouse). Plots of the mean distributions of duration and mean pitch of USVs produced by mice during different non-vocal behaviors revealed highly overlapping distributions for each age group (Figures 4B, C; *p* < 0.05 for the difference in median USV duration of P10 locomotion USVs vs. P10 lying still USVs; Wilcoxon signed-ranks test; *p* > 0.05 for differences in median values for all other duration comparisons and for all mean pitch comparisons). Distributions of other pre-defined acoustic features, including USV slope, frequency modulation index, and spectral purity, were also highly overlapping for USVs produced during different non-vocal behavior within each age group, albeit with a handful of significant but small differences in median values (Supplementary Figures 5, 6 and Supplementary Table 1).

Next, we examined and compared the acoustic features of USVs produced during different non-vocal behaviors within each age group using AVA. We assigned a non-vocal behavior code to each USV syllable and color-coded latent syllable representations according to the non-vocal behavior performed during the production of each USV (Figure 4D; lying still USVs, dark blue; locomotion USVs, light blue; wriggling USVs, red; grooming USVs, chartreuse). This analysis revealed substantial overlap in the latent syllable representations of USVs produced during different non-vocal behaviors within each age group. To quantify differences between these distributions, we calculated MMD between distributions of latent syllable representations to generate within age comparisons of USVs produced during different non-vocal behaviors (Supplementary Figure 7). MMD comparisons revealed that acoustic differences between USVs produced during different non-vocal behaviors tended to be either similar in magnitude or smaller than acoustic differences between USVs produced during the same non-vocal behaviors. These analyses, together with analyses of pre-defined acoustic features, support the conclusion that mouse pups produce USVs with highly overlapping distributions of acoustic features different categories of non-vocal behaviors.

### Relationship of USV rates to non-vocal movement intensity

**FIGURE 5:**
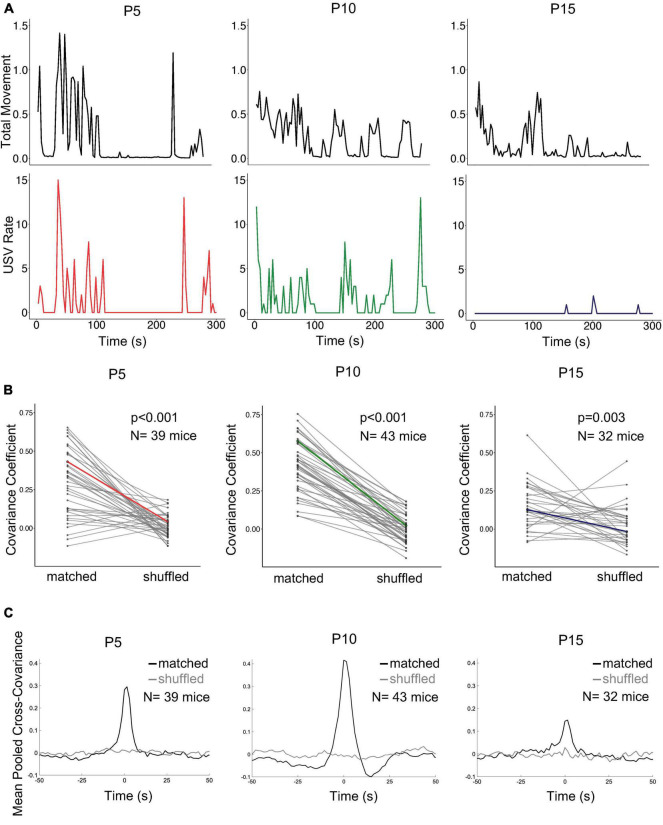
Relationship of USV rates to intensity of non-vocal movements. **(A)** Plots of non-vocal movement intensity (top row) and USV rate (bottom row) are shown for three representative trials. P5, left (*N* = 39); P10, middle (*N* = 43), P15, right (*N* = 32). **(B)** Covariance coefficients for matched and shuffled comparisons are plotted for each mouse in each postnatal age group. Colored lines correspond to the examples shown in (panel **A**). **(C)** Mean pooled matched (black) and shuffled (gray) cross-covariances are shown for the comparison of movement intensity and USV rate in the three postnatal age groups.

Although we found that rates of isolation USVs are related to categories of non-vocal behavior, these relationships were relatively weak (high variability in mean USVs per second of behavior for different pups, Figure 3A). Even within a given category of behavior, non-vocal movements are dynamic and vary in intensity from moment-to-moment, and we wondered whether dynamic variations in movement intensity might be related to dynamic variations in USV rate. To test this idea, movement intensity in each trial was quantified using Annolid software as the total movement in the instance mask of the pup across consecutive pairs of frames (includes locomotion as well as head and limb movements, see Methods; Figure 5A). We then calculated the normalized cross-covariance between movement intensity and USV rate for each trial (see Methods). Because each pup was recorded for 10 min, we separately considered the first 5 min and the second 5 min of recording to generate both matched and shuffled comparisons for each trial (Figures 5B, C; matched: first 5 min USVs vs. first 5 min movement, last 5 min USVs vs. last 5 min movement; shuffled: first 5 min USVs vs. last 5 min movement, last 5 min USVs vs. first 5 min movement). Mean matched and shuffled cross-covariances were pooled from pups of the same age to generate mean pooled comparisons for each age group (Figure 5C). To compare the strength of this covariance across and within different age groups, we then calculated mean pooled covariance coefficients (CC, see Methods) for matched and shuffled comparisons within each age. CCs were then calculated and compared for each trial’s matched and shuffled comparisons (see Methods; Figure 5B). CCs for matched comparisons were significantly higher than CCs for shuffled comparisons at all ages (Figure 5B; mean matched CC’s for P5: 0.29 ± 0.21; P10: 0.42 ± 0.17; P15: 0.15 ± 0.15; mean shuffled CC’s for P5: 0.01 ± 0.07; P10: 0.00 ± 0.09; P15: 0.03 ± 0.13; *p* < 0.005 for all ages, two-way ANOVA with repeated measures on one factor followed by *post-hoc* tests). We conclude that rates of isolation USVs are well-related on average to the intensity of non-vocal movements within individual trials, and this relationship is particularly pronounced at earlier postnatal ages.

### Relationship of USV acoustic features to non-vocal movement intensity

Finally, we considered whether mice produce USVs with different acoustic features as they engage in movements of different intensity. We note that USVs produced while lying still were highly overlapping in their acoustic features with USVs produced during active behaviors within each age group (Figure 4), suggesting that USV acoustic features are unlikely to be well-related to the intensity of non-vocal movements. Nonetheless, we formally considered this idea by color-coding latent syllable representations in AVA according to intensity of non-vocal movements that were ongoing at the time a given USV was produced (Supplementary Figure 8). As expected, this analysis revealed substantial overlap in the latent syllable representations of USVs produced during different intensities of non-vocal movements within each age group, indicating that non-vocal movement intensity cannot account for within age variability in the acoustic features of isolation USVs.

## Discussion

In this study, we compared the vocal and non-vocal behaviors of mouse pups over early postnatal development to test the hypothesis that within age variability in the rates and acoustic features of isolation USVs is related to the production of non-vocal behaviors. Consistent with previous studies (Liu et al., 2003; Grimsley et al., 2011; Yin et al., 2016; Castellucci et al., 2018), we found that rates of isolation USVs peak in P10 pups and then subsequently decline, paralleling the gradual increase in pups’ ability to locomote and thermoregulate independently (Theiler, 1972). Similarly, measurements of pre-defined features and machine learning-based analyses of USV acoustic features revealed dramatic changes over early postnatal development, consistent with previous studies (Liu et al., 2003; Grimsley et al., 2011; Yin et al., 2016; Castellucci et al., 2018). By comparing USV rates to the production of non-vocal behaviors within each trial, we determined that pups in all age groups (P5, P10, and P15) produce higher rates of isolation USVs during active behaviors compared to periods of inactivity. Moreover, rates of USV production were well-correlated with movement intensity in individual trials, particularly in P5 and P10 pups. In contrast, the acoustic features of isolation USVs were unrelated to the production of different categories and intensities of non-vocal movements, and this was true across all age groups. To our knowledge, this is the first study to investigate the possibility that rates and acoustic features of mouse pup isolation USVs may be related to non-vocal behaviors.

Why are pups more likely to produce isolation USVs during active behaviors than during periods of inactivity and particularly during high intensity movements? One factor contributing to these relationships could be changes in respiration associated with movement. As they transition from rest to vigorous movement, animals increase their respiratory rate (Bramble and Carrier, 1983; DiMarco et al., 1983; Mateika and Duffin, 1995; Gravel et al., 2007; Hérent et al., 2020) and switch from passive to active expiration (Abraham et al., 2002; Abdala et al., 2009), a process which recruits abdominal muscles to increase intrathoracic pressure. Although no study to our knowledge has measured respiratory rates or patterns during the production of different non-vocal behaviors in mouse pups, it is possible that movement-associated changes in respiration promote USV production. On the one hand, brainstem respiratory circuits (Pagliardini et al., 2011; Dutschmann and Dick, 2012; Ikeda et al., 2017; Yackle et al., 2017; Zuperku et al., 2017; Del Negro et al., 2018) might influence the activity of brainstem neurons known to be important for USV production in adults (Jürgens, 2002; Tschida et al., 2019; Hartmann and Brecht, 2020) and pups (Hernandez-Miranda et al., 2017; Wei et al., 2021). A related possibility is that neuronal circuits important for movement generation, some of which in turn contribute to movement-related changes in respiration (Eldridge et al., 1981; Gariépy et al., 2012), might act on brainstem vocal-respiratory circuits to promote pup USV production.

Movement could also promote USV production as a consequence of increased intrathoracic pressure during periods of active expiration. Some studies have suggested that pup USVs are produced as a passive, acoustic byproduct of laryngeal braking, a respiratory mechanism in which contraction of abdominal muscles and constriction of the larynx improves gas exchange in the lungs during periods of high metabolic demand (Gilfoil et al., 1959; Youmans et al., 1963; Blumberg and Alberts, 1990; Blumberg and Sokoloff, 2001); see also Shair and Jasper (2003). If laryngeal braking accounts for pup USV production in at least some instances, any factor that increases intrathoracic pressure, including the transition to active expiration during high intensity movements, might promote USV production. Finally, we note that if for any reason a pup is in a behavioral state that is favorable to USV production, movement-related increases in respiratory rates *per se* could lead to increased rates of USV production. Rodents produce USVs as they exhale (Roberts, 1972; Sirotin et al., 2014; Wei et al., 2021), and movement-related increases in the number of respiratory cycles per second would provide more opportunities for USV production to occur than when pups are at rest.

A second, non-mutually exclusive possibility is that movement-associated increases in arousal contribute to the observed relationships between non-vocal movements and USV rates. Previous studies have found that marmosets are more likely to produce contact calls during periods of heightened arousal, as measured by fluctuations in heart rate (Borjon et al., 2016; Gustison et al., 2019). Similarly, humans exhibit increases in heart rate and blood pressure prior to speech onset (Lynch et al., 1980; Linden, 1987). We did not include any physiological measures of arousal in the current study, but such measures could be included in future work to better understand how these variables relate to pup USV production and to what extent they can account for the relationship of non-vocal movements to pup USV rates within trials, across trials, and across early postnatal development.

While rates of isolation USVs were on average well-related to movement intensity in P5 and P10 pups, the strength of this relationship decreased in P15 mice (Figure 5). This finding is perhaps not surprising, given that rates of isolation USVs decline as pups gain the ability to thermoregulate (Okon, 1970a; Hahn et al., 1997; Thornton et al., 2005), with isolation USV rates reaching near-zero around or shortly after 2 weeks of age (Figure 1B; Liu et al., 2003; Grimsley et al., 2011; Yin et al., 2016; Castellucci et al., 2018). Interestingly, a strong relationship between USV rates and non-vocal movements re-emerges in adult rodents. Specifically, rates of 50 kHz USVs produced by adult rats are tightly coupled at the sub-second timescale to locomotion speed, both during and outside of social interactions (Laplagne and Elías Costa, 2016). Conversely, adult rats produce 22 kHz USVs almost exclusively while immobile (Wöhr et al., 2005; Wöhr and Schwarting, 2008; Laplagne and Elías Costa, 2016). Although adult mice produce only low rates of USVs in the absence of social partners or cues, adult males and females increase production of 70 kHz USVs during chases (Neunuebel et al., 2015; Sangiamo et al., 2020).

Similar to what has been described in adult rodents (Laplagne and Elías Costa, 2016), however, we found that the relationship between non-vocal movements and pup USV production is not obligatory. That is to say that pups can produce USVs while lying still and conversely, pups can produce non-vocal movements without vocalizing (Figures 3–5A, Supplementary Figure 4). In addition, we note that there is trial-to-trial variability in the strength of the relationship between isolation USV rates and movement intensity (Figure 5B). Taken together, our findings indicate that rates of isolation USVs are related to both the category and intensity of non-vocal movements, but additional factors must also contribute to within age variability in pup USV production. These factors are likely to be state-like variables rather than stable traits, given that rates of USVs produced by an individual pup are variable across multiple same-day recordings (Barnes et al., 2017) and recordings performed on consecutive days (Rieger and Dougherty, 2016).

Although we uncovered clear relationships between non-vocal behaviors and USV rates, no such relationships were revealed in our comparisons of non-vocal behaviors and USV acoustic features. Previous studies in adult rodents have suggested that there are differences in the acoustic features of USVs produced during different non-vocal behaviors. For example, adult male mice produce USVs that are longer, lower frequency, and more harmonic during mounting as compared to USVs produced during social investigation (Hanson and Hurley, 2012; Matsumoto and Okanoya, 2016). In addition, adult male mice produce USVs that differ in acoustic features while interacting with social cues (e.g., urine) as compared to an anesthetized or live social partner (Chabout et al., 2015) and when interacting with a social partner as compared to after the social partner departs (Hanson and Hurley, 2012; Yang et al., 2013). Although not all of these studies explicitly considered or quantified non-vocal behaviors, undoubtedly, mice engage in different types and intensities of non-vocal movements in these different social contexts. In addition, a previous study concluded that acoustic features of prairie vole USVs covary with heart rate (Stewart et al., 2015), although we note that the conclusions of this study were based on only 65 USVs produced by 3 voles.

Nonetheless, in the current study, we considered many thousands of USVs produced by 45 mouse pups and found that USVs produced during different categories of non-vocal behavior did not differ substantially in their acoustic features, nor did USV acoustic features vary according to the intensity of non-vocal movements produced concurrently with vocalization. Although we found subtle differences in distributions of the duration, mean pitch, and other acoustic features of USVs produced during different non-vocal behaviors, distributions of these pre-defined acoustic features demonstrated considerable variability and were largely overlapping for USVs produced during different non-vocal behaviors (Figures 4A–C, Supplementary Figures 5, 6). Our findings with AVA further support the conclusion that mouse pups do not produce acoustically distinct types of USVs during different categories of non-vocal behaviors (Figure 4D) or during different intensities of non-vocal movements (Supplementary Figure 8). Ultimately, the acoustic features of a given USV depend on respiratory rate, subglottal pressure and air flow, laryngeal muscle activation, and vocal tract configuration (Riede, 2011, 2013, 2018; Mahrt et al., 2016; Hülsmann et al., 2019; Håkansson et al., 2022). Thus, our findings suggest that patterns of activity in the premotor and motor neurons that control the vocal actuators are not highly constrained by pup non-vocal behavior. The factors that contribute to variability in the acoustic features of pup and adult USVs, in terms of both moment-to-moment variability within a given behavioral context as well as differences across contexts, are an important topic that warrants further study.

Previous studies have found that rates of isolation USV production are critical for eliciting maternal retrieval. For example, anesthetized pups are retrieved at lower rates than vocalizing pups (Uematsu et al., 2007), more vocal pups within a litter are retrieved more quickly (Bowers et al., 2013), and pups with neural circuit alterations that cannot produce USVs receive lower levels of maternal care (Hernandez-Miranda et al., 2017). Similarly, temporal features of isolation USVs (duration and inter-syllable interval) are critical for maternal responses (Ehret and Haack, 1982; Ehret, 1992; Uematsu et al., 2007; Schiavo et al., 2020). On the other hand, the contribution of isolation USV spectral features to pup survival is less clear. Lactating females approach playbacks of ultrasonic white noise and ultrasonic tone bursts at rates that are comparable to responses to playback of unaltered isolation USVs, as long as the temporal organization of synthetic stimuli is matched to that of natural isolation USVs (Ehret and Haack, 1982; Ehret, 1992; Uematsu et al., 2007). Nonetheless, both rates and acoustic features of pup USVs are commonly characterized in mouse models of neurodevelopmental disorders to assess differences in communication, without any accompanying consideration of non-vocal behaviors (Scattoni et al., 2009; Wöhr et al., 2011, 2013; Ey et al., 2013; Wöhr, 2014, 2015). Our finding that rates of isolation USVs are well-related to the intensity of ongoing non-vocal movements suggests that careful characterization of non-vocal behaviors alongside measurements of isolation USVs will yield a richer understanding of behavioral differences in mouse models of communication disorders.

## Data availability statement

All source data generated in this study as well as custom Matlab codes for USV detection have been deposited in a digital data repository and can be accessed at https://doi.org/10.7298/hck0-e345. Autoencoded Vocal Analysis (Goffinet et al., 2021) is freely available online: https://github.com/jackgoffinet/autoencodedvocal-analysis. Annolid is open-source and can be obtained from the GitHub repository https://github.com/cplab/annolid. The labeled dataset and trained instance segmentation model are also be included in the digital data repository. Further information and requests for resources should be directed to the corresponding author, KT (kat227@cornell.edu).

## Ethics statement

The animal study was reviewed and approved by Cornell University Institutional Animal Care and Use Committee (protocol #2020-001).

## Author contributions

NP and KT designed the experiments and wrote the manuscript. NP conducted the experiments. All authors analyzed the data and approved the final version.

## Footnotes

1
https://cplab.science/annolid
