# Supplementary material for: Rates of ultrasonic vocalizations are more strongly related than acoustic features to non-vocal behaviors in mouse pups
Source: Front Behav Neurosci. 2022 Dec 19;16:1015484. doi: 10.3389/fnbeh.2022.1015484 (PMC9805956; doi:10.3389/fnbeh.2022.1015484)
Supplement: Supplementary file 5 [file Data_Sheet_1.PDF]

Supporting Information

Figures S1-8

Movies S1-4

Tables S1-2

Figure S1

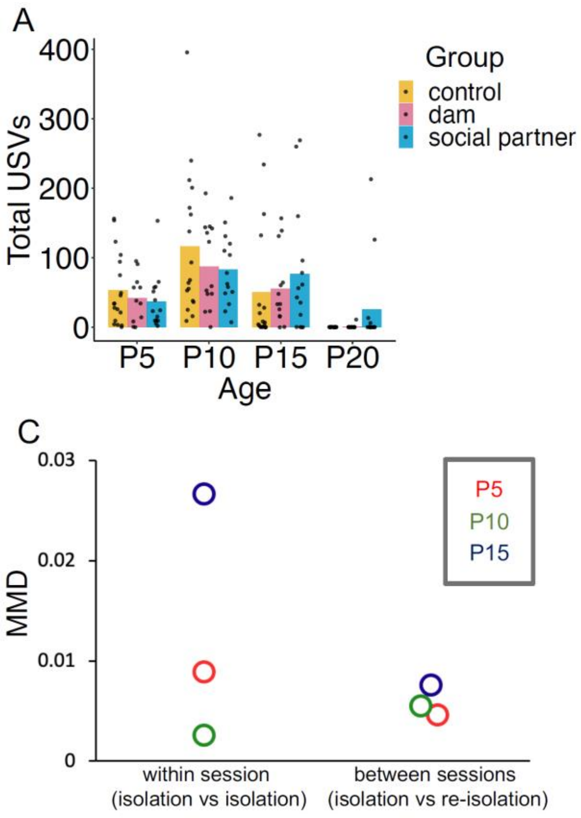

A
Total USVs
400
300
200
100
0
P5
P10
P15
P20
Age
Group
control
dam
social partner
B
Isolation
Re-isolation
n = 12914 USVs
n = 8708 USVs
C
MMD
0.03
0.02
0.01
0
P5
P10
P15
within session
(isolation vs isolation)
between sessions
(isolation vs re-isolation)
D
MMD
0.1
0.075
0.05
0.025
0
P5-P15
P10-P15
P5-P10
within age
between ages

Figure S1. Further quantification of rates and acoustic features of USVs produced during isolation and re-isolation sessions. (A) Number of USVs recorded during re-isolation sessions at P5, P10, P15 and P20. In the immediately preceding 5-minute social session, pups were recorded either with their dam, with a novel adult female, or with no social partner (control). No significant differences in rates of re-isolation USVs were found between social groups at any age ($p > 0.05$ for all within age comparisons). (B) UMAP projections of latent syllable representations of USVs produced by P5, P10, and P15 mice during isolation (gray points) and re-isolation sessions (black points). Points represent individual syllables and are closer to each other if acoustically more similar. (C) Maximum Mean Discrepancy (MMD) was calculated between distributions of latent syllable representations to generate two comparisons for each age: isolation vs. isolation (within session) and isolation vs. re-isolation (between sessions). To generate within session comparisons, isolation sessions of different animals randomly assigned to one of two groups were compared. Comparisons with higher MMD values are more dissimilar. (D) MMD values were calculated between distributions of latent syllable representations to compare differences in acoustic features of isolation USVs within ages and between ages. Comparisons with higher MMD values are more dissimilar. Within age comparisons were generated by considering USVs produced during isolation sessions vs. USVs produced during re-isolation sessions and calculating MMD between these two distributions.

Figure S2

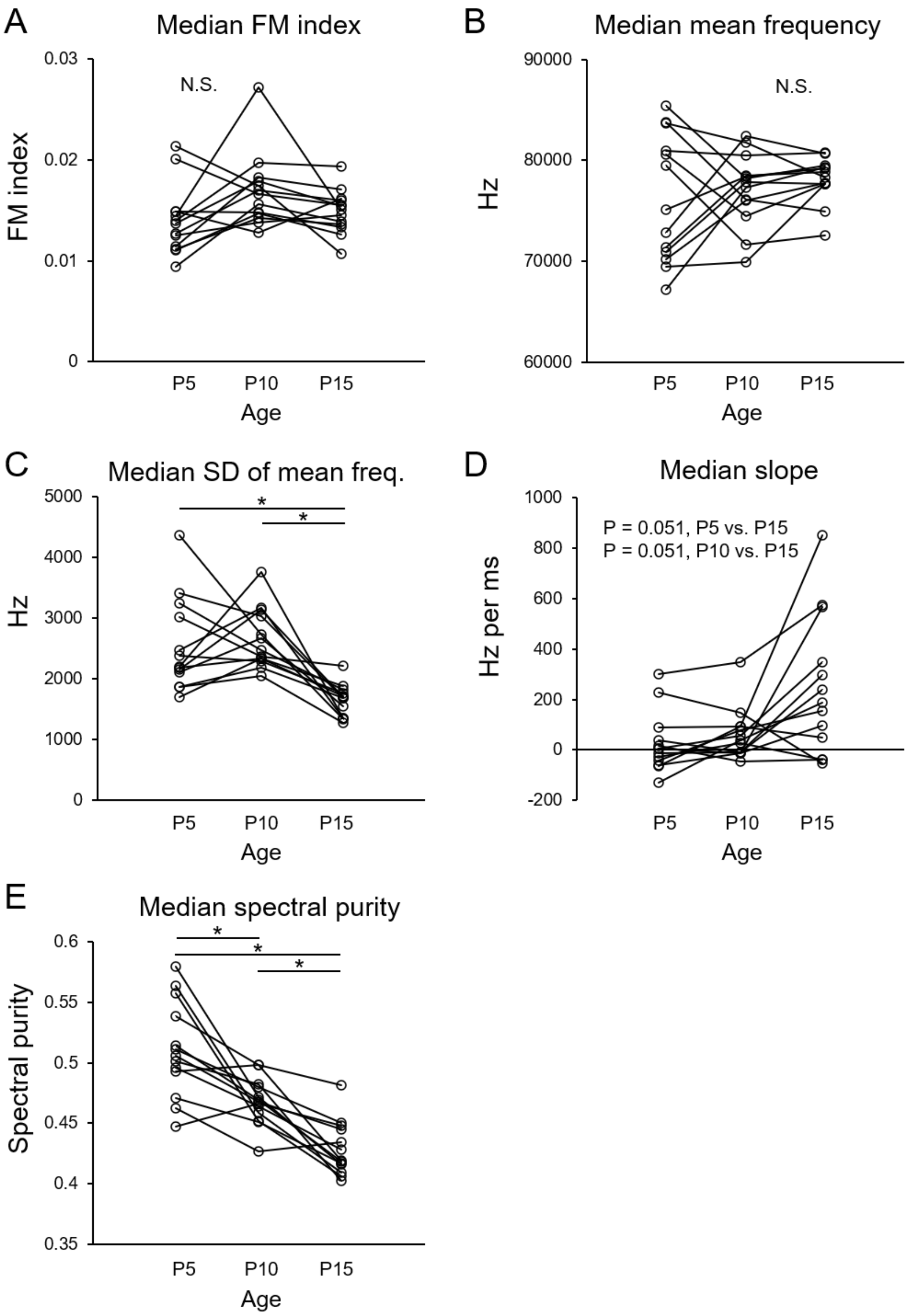
A
Median FM index
N.S.
FM index
0.03
0.02
0.01
0
P5
P10
P15
Age
B
Median mean frequency
N.S.
Hz
90000
80000
70000
60000
P5
P10
P15
Age
C
Median SD of mean freq.
*
*
Hz
5000
4000
3000
2000
1000
0
P5
P10
P15
Age
D
Median slope
P = 0.051, P5 vs. P15
P = 0.051, P10 vs. P15
Hz per ms
1000
800
600
400
200
0
-200
P5
P10
P15
Age
E
Median spectral purity
*
*
*
Spectral purity
0.6
0.55
0.5
0.45
0.4
0.35
P5
P10
P15
Age

Figure S2. Quantification of additional pre-defined USV acoustic features across age groups. (A) Median values of USV frequency modulation (FM) index vs. age are plotted for N = 13 mice that produced > 50 USVs at P5, P10, and P15 (mean median FM index for P5 mice: 0.014 ± 0.003; P10: 0.017 ± 0.003; P15: 0.015 ± 0.002; no significant differences). (B) Same, for USV mean frequency (mean median mean frequency for P5 mice: 76220 ± 6315; P10: 77158 ± 3616; P15: 78058 ± 2234; no significant differences). (C) Same, for the standard deviation of mean USV frequency (mean median standard deviation of mean frequency for P5 mice: 2533 ± 769; P10: 2653 ± 496; P15: 1634 ± 265; $p < 0.05$ for P5 vs. P15 and for P10 vs. P15; $p < 0.05$ for P5 vs. P15 and for P10 vs. P15). (D) Same, for USV slope (mean median slope for P5 mice: 26.3 ± 119.5; P10: 60.5 ± 102.6; P15: 248.7 ± 276.0; no significant differences). (E) Same, for USV spectral purity (mean median spectral purity for P5 mice: 0.51 ± 0.04; P10: 0.47 ± 0.02; P15: 0.42 ± 0.02; $p < 0.05$ for all comparisons). See Methods for additional details of pre-defined acoustic feature calculation and see Table S1 for statistics details of these comparisons. All comparisons were analyzed with Friedman tests, followed by post-hoc tests as warranted.

Figure S3

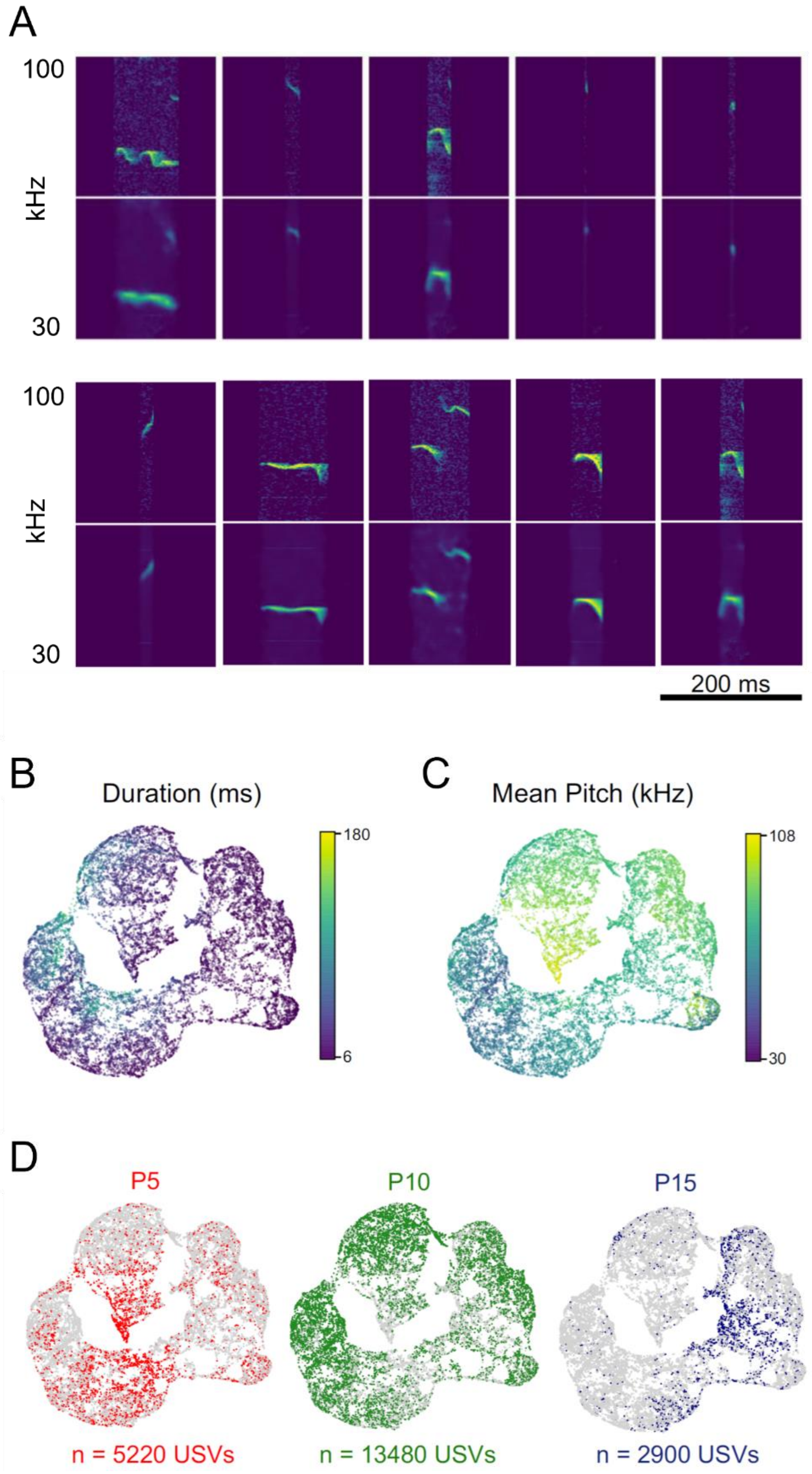
A
100
kHz
30
100
kHz
30
200 ms
B
Duration (ms)
180
6
C
Mean Pitch (kHz)
108
30
D
P5
P10
P15
n = 5220 USVs
n = 13480 USVs
n = 2900 USVs

Figure S3. Reconstructed spectrograms of isolation USVs produced by P5, P10, and P15 mice and UMAP projections of latent syllable representations, color-coded by hand-picked acoustic features. (A) Reconstructed spectrograms of representative syllables. (B) UMAP projections of syllable representations are color-coded by duration (ms). (C) UMAP projections of syllable representations are color-coded by mean pitch (kHz). (D) UMAP projections of syllable representations color-coded by age (P5, red; P10, green; P15, blue) are reproduced from Figure 1D for comparison.

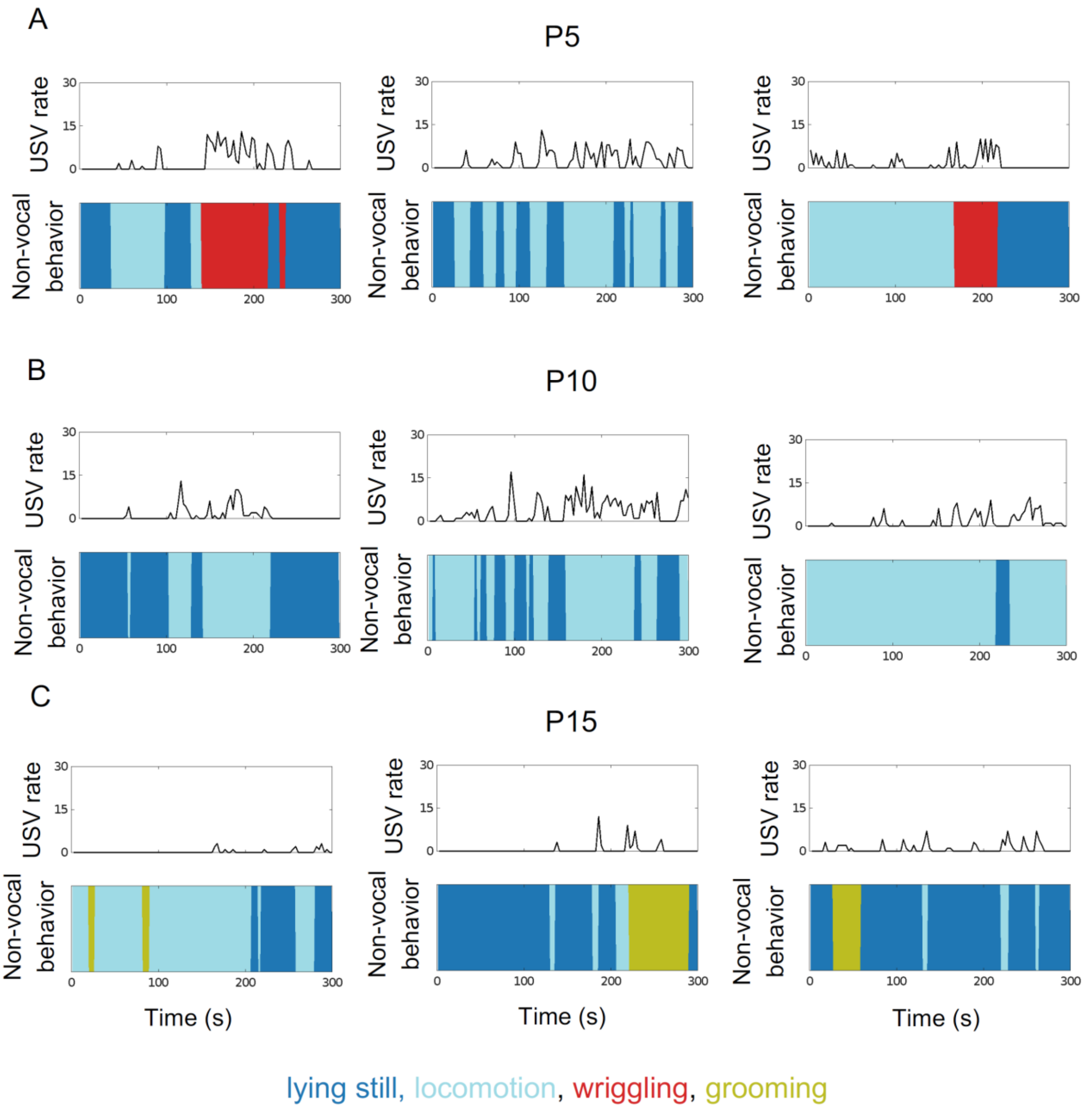

Figure S4. Ethograms of USV production and non-vocal behavior. Representative ethograms are shown for trials from P5 mice (A), P10 mice (B) and P15 mice (C). The top half of each plot shows USV rate over time (total USVs in each 3s-long bin), and the bottom half of each plot shows the occurrence of non-vocal behaviors across time (lying still, dark blue; locomotion, light blue; wriggling, red; grooming: chartreuse).

Figure S5

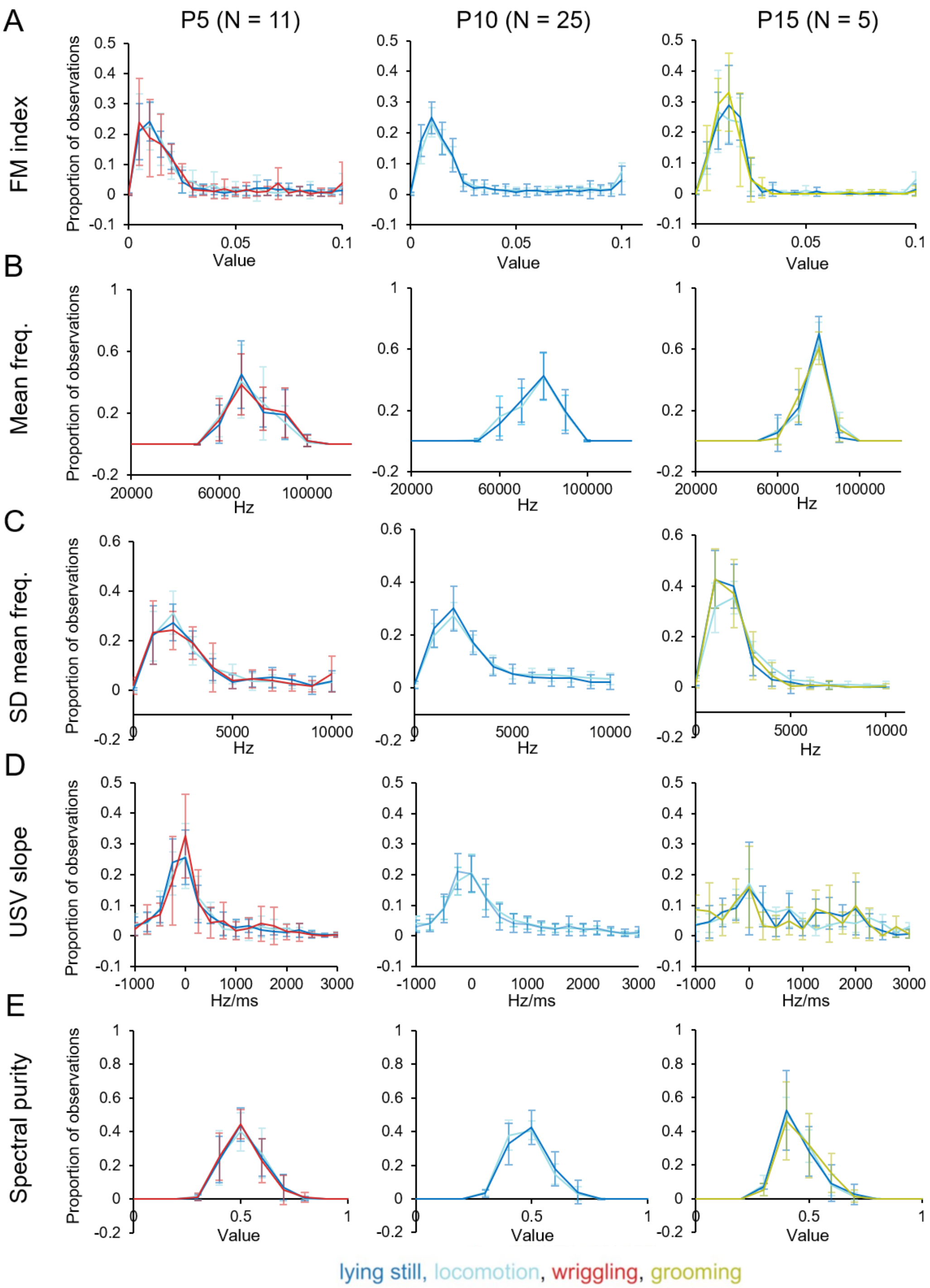
A
B
C
D
E
P5 (N = 11)
P10 (N = 25)
P15 (N = 5)
FM index
Mean freq.
SD mean freq.
USV slope
Spectral purity
Proportion of observations
Value
Hz
Hz/ms
lying still, locomotion, wriggling, grooming

Figure S5. Distributions of additional pre-defined acoustic features for USVs produced during different non-vocal behaviors. (A) The distributions of frequency modulation (FM) index of isolation USVs produced during different non-vocal behaviors is shown for P5 (left, N = 11), P10 (middle, N = 25), and P15 mice (right, N = 5). P10 mice produced < 50 total USVs during wriggling, so P10 wriggling USVs are excluded from analysis. (B) Same, for USV mean frequency. (C) Same, for the standard deviation of mean USV frequency. (D) Same, for USV slope. (E) Same, for USV spectral purity. See Methods for additional details of pre-defined acoustic feature calculation.

Figure S6

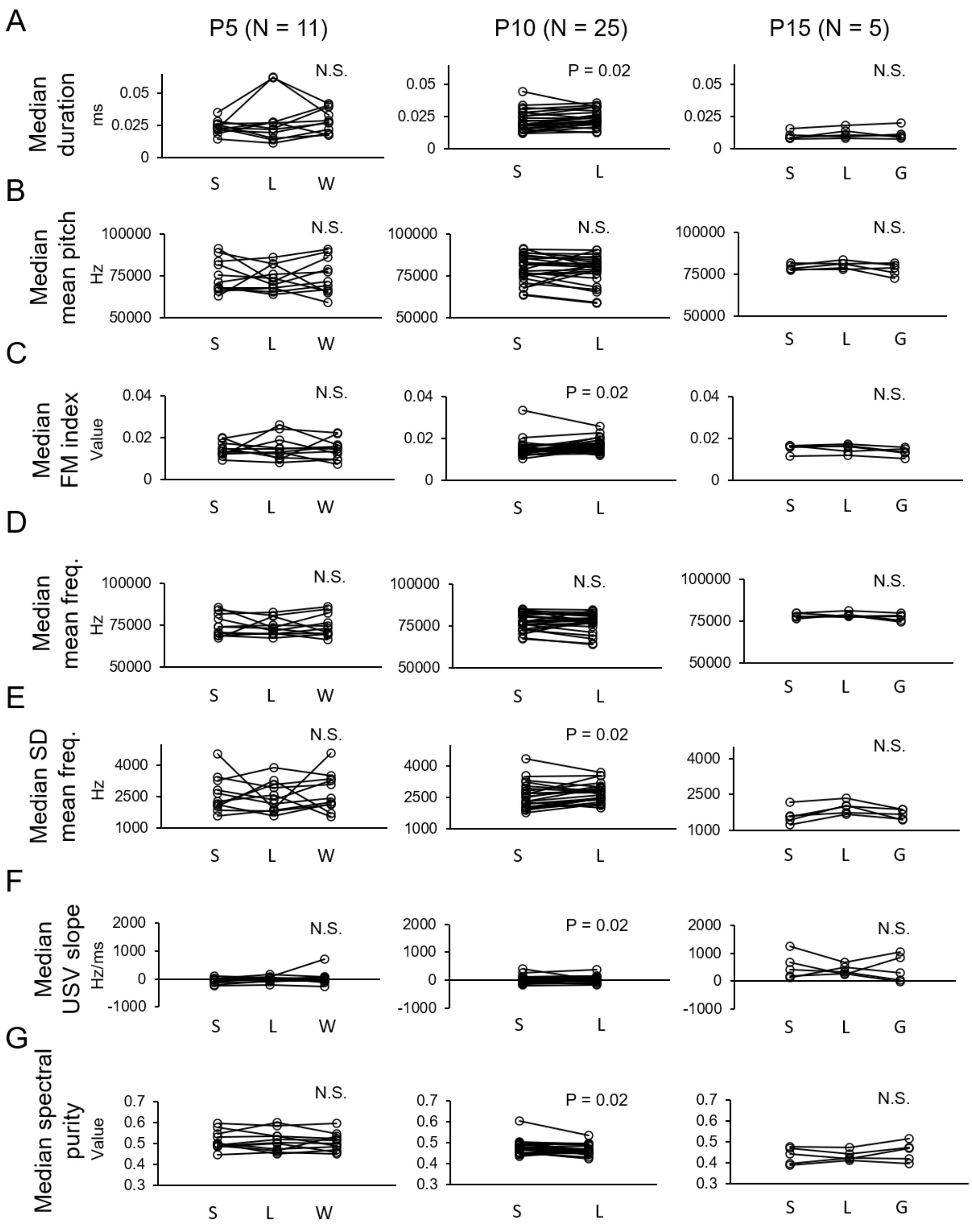
A
B
C
D
E
F
G
P5 (N = 11)
P10 (N = 25)
P15 (N = 5)
Median duration
ms
Median mean pitch
Hz
Median FM index
Value
Median mean freq.
Hz
Median SD mean freq.
Hz
Median USV slope
Hz/ms
Median spectral purity
Value
N.S.
P = 0.02
S
L
W
G

Figure S6. Median values of pre-defined acoustic features for USVs produced during different non-vocal behaviors. (A) Median values of USV duration are plotted for mice that produced > 10 USVs during each non-vocal behavior for each age group (mean median duration for P5 lying still: 0.024 ± 0.003; P5 locomotion: 0.029 ± 0.019; P5 wriggling: 0.024 ± 0.007; P10 lying still: 0.021 ± 0.008; P10 locomotion: 0.024 ± 0.006; P15 lying still: 0.010 ± 0.003; P15 locomotion: 0.011 ± 0.005; P15 grooming: 0.011 ± 0.005; $p < 0.05$ for P10 lying still vs. locomotion). (B) Same, for mean pitch (mean median mean pitch for P5 lying still: 73978 ± 11820; P5 locomotion: 76820 ± 9445; P5 wriggling: 74849 ± 14590; P10 lying still: 79035 ± 8164; P10 locomotion: 78360 ± 8666; P15 lying still: 79091 ± 1792; P15 locomotion: 80435 ± 2202; P15 grooming: 77905 ± 3696; no significant differences). (C) Same, for FM index (mean median FM index for P5 lying still: 0.013 ± 0.003; P5 locomotion: 0.016 ± 0.007; P5 wriggling: 0.014 ± 0.003; P10 lying still: 0.015 ± 0.004; P10 locomotion: 0.017 ± 0.003; P15 lying still: 0.016 ± 0.002; P15 locomotion: 0.015 ± 0.002; P15 grooming: 0.014 ± 0.002; $p < 0.05$ for P10 lying still vs. locomotion). (D) Same, for mean frequency (mean median mean frequency for P5 lying still: 74134 ± 8157; P5 locomotion: 76232 ± 6879; P5 wriggling: 75506 ± 9199; P10 lying still: 77539 ± 5258; P10 locomotion: 77129 ± 5763; P15 lying still: 78133 ± 1483; P15 locomotion: 78711 ± 1336; P15 grooming: 77174 ± 2187; no significant differences). (E) Same, for the standard deviation of mean USV frequency (mean median SD of mean frequency for P5 lying still: 2208 ± 371; P5 locomotion: 2611 ± 574; P5 wriggling: 2382 ± 853; P10 lying still: 2511 ± 612; P10 locomotion: 2696 ± 450; P15 lying still: 1606 ± 347; P15 locomotion: 1950 ± 270; P15 grooming: 1671 ± 219; $p < 0.05$ for P10 lying still vs. locomotion). (F) Same, for USV slope (mean median slope for P5 lying still: -71.0 ± 102.4; P5 locomotion: -12.6 ± 134.5; P5 wriggling: -58.8 ± 137.7; P10 lying still: 9.4 ± 130.8; P10 locomotion: 41.5 ± 107.4; P15 lying still: 528.6 ± 450.7; P15 locomotion: 402.1 ± 187.7; P15 grooming: 450.4 ± 484.7; $p < 0.05$ for P10 lying still vs. locomotion). (G) Same, for USV spectral purity (mean median spectral purity for P5 lying still: 0.54 ± 0.05; P5 locomotion: 0.52 ± 0.05; P5 wriggling: 0.51 ± 0.06; P10 lying still: 0.48 ±

0.03; P10 locomotion: 0.47 ± 0.02; P15 lying still: 0.44 ± 0.04; P15 locomotion: 0.44 ± 0.02; P15 grooming: 0.46 ± 0.05; $p < 0.05$ for P10 lying still vs. locomotion). P5 and P15 comparisons were analyzed with Friedman tests, followed by post-hoc tests as warranted, and P10 comparisons were analyzed with Wilcoxon signed-rank tests. See Methods for additional details of pre-defined acoustic feature calculation and see Table S1 for statistics details of these comparisons. S = lying still; L = locomotion; W = wriggling; G = grooming.

Figure S7

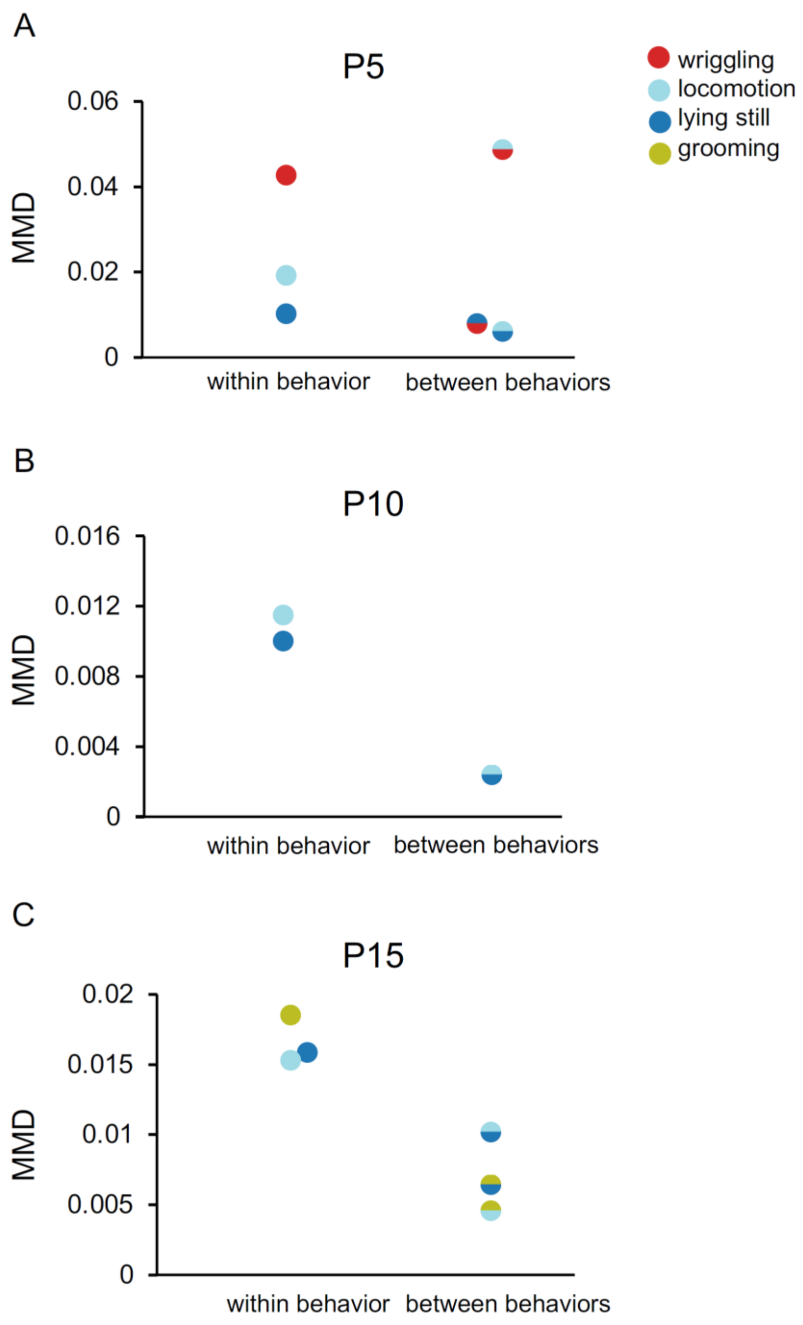
A
P5
wriggling
locomotion
lying still
grooming
0.06
0.04
0.02
0
MMD
within behavior
between behaviors
B
P10
0.016
0.012
0.008
0.004
0
MMD
within behavior
between behaviors
C
P15
0.02
0.015
0.01
0.005
0
MMD
within behavior
between behaviors

Figure S7. Maximum Mean Discrepancy between distributions of latent representations of isolation USVs produced during different categories of non-vocal behavior. (A) MMD values were calculated between distributions of latent syllable representations to compare differences in acoustic features of isolation USVs produced during different non-vocal behaviors in P5 pups. (B) Same as (A), for P10 USVs. (C) Same as (A), for P15 USVs. Comparisons with higher MMD values are more dissimilar.

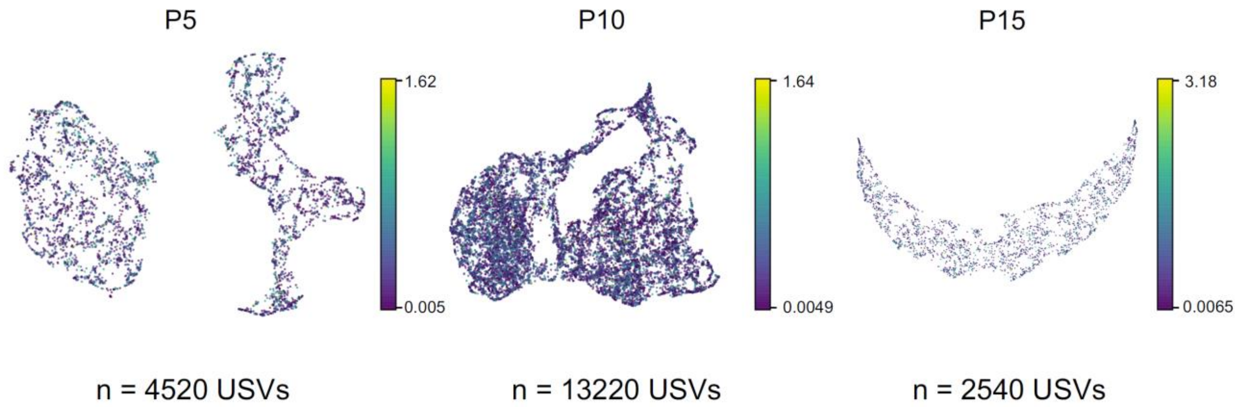

Figure S8. Relationship of USV acoustic features to intensity of non-vocal movements. UMAP projections of latent descriptions of USV syllables produced by P5 (left), P10 (middle) and P15 mice (right). Individual syllable representations are outlined in color according to the intensity of non-vocal movement that occurred while that syllable was produced. Please note that the by-age bokeh plots differ in shape from those that appear in Fig. 4D because the VAE was trained independently for these two analyses, generates different models, and therefore generates different latent representations of USV syllables, even when trained on the same inputs.

Movie S1. Example of USVs produced during wriggling. Representative video (top) of a P5 pup producing USVs during wriggling. Bottom panel shows a spectrogram of USVs synchronized to the video, and the audio is pitch shifted (80 kHz to 5 kHz) to a range audible to humans.

Movie S2. Example of USVs produced during locomotion. Representative video (top) of a P10 pup producing USVs while engaged in locomotion. Bottom panel shows a spectrogram of USVs synchronized to the video, and the audio is pitch shifted (80 kHz to 5 kHz) to a range audible to humans.

Movie S3. Example of USVs produced while lying still. Representative video (top) of a P10 pup producing USVs while lying still. Bottom panel shows a spectrogram of USVs synchronized to the video, and the audio is pitch shifted (80 kHz to 5 kHz) to a range audible to humans.

Movie S4. Example of USVs produced while grooming. Representative video (top) of a P15 pup producing USVs during grooming. Bottom panel shows a spectrogram of USVs synchronized to the video, and the audio is pitch shifted (80 kHz to 5 kHz) to a range audible to humans.

Table S1. Statistical summary. Details of statistical analyses used in this study are presented.

Figure	Comparison	Tests	Test statistics	Outcome	Notes
Fig. 1B	USV counts x age *N = 42 mice*	One-way repeated measures ANOVA; post-hoc paired t-tests with Bonferroni corrections	F = 50.43 Degrees of freedom for age = 1.63 Degrees of freedom for residuals = 66.95	p < 0.001 (main effect of age) ** p < 0.001 (P5 vs. P10) ** p = 0.02 (P5 vs. P15) * p < 0.001 (P5 vs. P20) ** p < 0.001 (P10 vs. P15) ** p < 0.001 (P10 vs. P20) ** p < 0.001 (P15 vs. P20) **	
Fig. 1C, left	Median USV duration x age *N = 13 mice*	Friedman, post-hoc Wilcoxon-signed-rank tests with Bonferroni corrections	Friedman's statistic = 18.5 Degrees of freedom for age = 2	p < 0.001 (main effect of age) ** p = 1.00 (P5 vs. P10) p = 0.008 (P5 vs. P15) ** p < 0.001 (P10 vs. P15) **	P20 excluded
Fig. 1C, middle	Median ISI x age *N = 7 mice*	Friedman, post-hoc Wilcoxon-signed-rank tests with Bonferroni corrections	Friedman's statistic = 11.1 Degrees of freedom for age = 2	p = 0.004 (main effect of age) ** p = 0.05 (P5 vs. P10) * p = 0.05 (P5 vs. P15) * p = 0.33 (P10 vs. P15)	P20 excluded

Fig. 2	Proportion time spent performing non-vocal behaviors x age *N = 40 mice*	2-way ANOVA with repeated measures on both factors; post-hoc paired t-tests with Bonferroni corrections	$F_{age} < 0.001$ $F_{behavior} = 751.94$ $F_{agexbehavior} = 44.51$ Degrees of freedom for age = 2 Degrees of freedom for behavior = 3 Degrees of freedom for age x behavior interaction= 6 Degrees of freedom for residuals for main effect of age = 78 Degrees of freedom for residuals for main effect of behavior = 117 Degrees of freedom for residuals for age x behavior interaction = 234	p = 1.00 (main effect of age) p < 0.001 (main effect of non-vocal behavior) ** p < 0.001 (interaction effect between non-vocal behavior vs. age) ** p < 0.001 (P5 vs. P10 wriggling) ** p < 0.001 (P5 vs. P15 wriggling) ** p = 0.585 (P10 vs. P15 wriggling) p < 0.001 (P5 vs. P10 locomotion) ** p < 0.001 (P5 vs. P15 locomotion) ** p = 0.19 (P10 vs. P15 locomotion) p < 0.001 (P5 vs. P10 lying still) ** p < 0.001 (P5 vs. P15 lying still) ** p = 0.19 (P10 vs. P15 lying still)	

				p = 1.00 (P5 vs. P10 grooming)<br>p < 0.001 (P5 vs. P15 grooming) **<br>p < 0.001 (P10 vs. P15 grooming) **	
Fig. 3A (left)	USVs produced per second of each behavior at P5<br>*N = 42 P5 mice*	Friedman, post-hoc Wilcoxon-signed-rank tests with Bonferroni corrections	Friedman's statistic = 6.69<br>Degrees of freedom for behavior = 2	p = 0.04 (main effect of non-vocal behavior) *<br>p = 0.01 (locomotion vs. lying still) *<br>p = 0.55 (locomotion vs. wriggling)<br>p = 0.01 (lying still vs. wriggling) *	No grooming USVs at P5
Fig. 3A (middle)	USVs produced per second of each behavior at P10<br>*N = 44 P10 mice*	Friedman, post-hoc Wilcoxon-signed-rank tests with Bonferroni corrections	Friedman's statistic = 84<br>Degrees of freedom for behavior = 2	p < 0.001 (main effect of non-vocal behavior) **<br>p < 0.001 (locomotion vs. lying still) **<br>p < 0.001 (locomotion vs. wriggling) **<br>p < 0.001 (lying still vs. wriggling) **	No grooming USVs at P10

Fig. 3A (right)	USVs produced per second of each behavior at P15<br>*N = 44 P15 mice*	Friedman, post-hoc Wilcoxon-signed-rank tests with Bonferroni corrections	Friedman's statistic = 21.9<br>Degrees of freedom for behavior = 2	$p < 0.001$ (main effect of non-vocal behavior) **<br>$p < 0.001$ (locomotion vs. lying still) **<br>$p < 0.001$ (locomotion vs. grooming) **<br>$p = 0.04$ (lying still vs. grooming) *	No wriggling USVs at P15
Fig. 4B (left)	Median USV duration x non-vocal behavior at P5<br>*N = 11 P5 mice*	Friedman, post-hoc Wilcoxon-signed-rank tests with Bonferroni corrections	Friedman's statistic = 3.45<br>Degrees of freedom for behavior = 2	$p = 0.18$ (main effect of non-vocal behavior)	No grooming USVs at P5
Fig. 4B (middle)	Median USV duration x non-vocal behavior at P10<br>*N = 25 P10 mice*	Wilcoxon-signed-rank test	Degrees of freedom for behavior = 1	$p = 0.015$ (locomotion vs. lying still) *	Very few wriggling USVs at P10 (N=41), excluded from analysis; no grooming USVs at P10

Fig. 4B (right)	Median USV duration x non-vocal behavior at P15 *N = 5 P15 mice*	Friedman, post-hoc Wilcoxon-signed-rank tests with Bonferroni corrections	Friedman's statistic = 3.89<br>Degrees of freedom for behavior = 2	$p = 0.14$ (main effect of non-vocal behavior)	No wriggling USVs at P15
Fig. 4C (left)	Median USV mean pitch x non-vocal behavior at P5 *N = 11 P5 mice*	Friedman, post-hoc Wilcoxon-signed-rank tests with Bonferroni corrections	Friedman's statistic = 2.36<br>Degrees of freedom for behavior = 2	$p = 0.31$ (main effect of non-vocal behavior)	No grooming USVs at P5
Fig. 4C (middle)	Median USV mean pitch x non-vocal behavior at P10 *N = 25 P10 mice*	Wilcoxon-signed-rank test	Degrees of freedom for behavior = 1	$p = 0.24$ (locomotion vs. lying still)	Very few wriggling USVs at P10 (41), excluded from analysis; no grooming USVs at P10
Fig. 4C (right)	Median USV mean pitch x non-vocal behavior at P15 *N = 5 P15 mice*	Friedman, post-hoc Wilcoxon-signed-rank tests with Bonferroni corrections	Friedman's statistic = 0.4<br>Degrees of freedom for behavior = 2	$p = 0.82$ (main effect of non-vocal behavior)	No wriggling USVs at P15
Fig. 5B	Matched and shuffled comparisons of mean pooled covariance coefficients	2-way ANOVA with repeated measures on one factor; post-hoc t-tests with Bonferroni corrections	$F_{age} = 12.54$<br>$F_{comparison\ type} = 192.17$<br>$F_{age x comparison\ type} = 18.21$<br>Degrees of freedom for age = 2	$p < 0.001$ (main effect of age) **<br>$p < 0.001$ (main effect of comparison type) **	

	across and within ages<br>*N = 39 P5 mice*<br>*N = 43 P10 mice*<br>*N = 32 P15 mice*		Degrees of freedom for comparison type = 1<br>Degrees of freedom for age x comparison type interaction= 2<br>Degrees of freedom for residuals = 111	$p < 0.001$ (interaction effect between comparison type vs. age) **<br>$p = 0.01$ (P5 vs. P10 matched) *<br>$p = 0.003$ (P5 vs. P15 matched) **<br>$p < 0.001$ (P10 vs. P15 matched) **<br>$p = 1.00$ (P5 vs. P10 shuffled)<br>$p = 1.00$ (P5 vs. P15 shuffled)<br>$p = 0.78$ (P10 vs. P15 shuffled)	
Fig. S1A	Re-isolation USV counts x age x group (control, social partner, or dam)<br>*P5 (N = 18 control,*<br>*N = 11 dam,*<br>*N = 14 sp);*<br>*P10 (N = 17 control,*<br>*N = 13 dam,*<br>*N = 14 sp);*<br>*P15 (N = 18 control,*	2-way ANOVA	$F_{age} = 48.72$<br>$F_{group} = 0.09$<br>$F_{agexgroup} = 0.30$<br>Degrees of freedom for age = 3<br>Degrees of freedom for group = 2<br>Degrees of freedom for age x group interaction= 6<br>Degrees of freedom for residuals = 165	$p < 0.001$ (main effect of age) **<br>$p = 0.92$ (main effect of group)<br>$p = 0.94$ (interaction effect between age vs. group)	

	N = 13 dam, N = 14 sp); P20 (N = 18 control, N = 13 dam, N = 14 sp)				
Fig. S2A	Median USV FM index x age *N = 13 mice*	Friedman, post-hoc Wilcoxon-signed-rank tests with Bonferroni corrections	Friedman's statistic = 6.62<br>Degrees of freedom for age = 2	p =0.04 (main effect of age) *<br>p = 0.12 (P5 vs. P10)<br>p = 0.65 (P5 vs. P15)<br>p = 0.08 (P10 vs. P15)	P20 excluded
Fig. S2B	Median mean USV frequency x age *N = 13 mice*	Friedman, post-hoc Wilcoxon-signed-rank tests with Bonferroni corrections	Friedman's statistic = 1.08<br>Degrees of freedom for age = 2	p = 0.58 (main effect of age)	P20 excluded
Fig. S2C	Median standard deviation of USV mean frequency x age *N = 13 mice*	Friedman, post-hoc Wilcoxon-signed-rank tests with Bonferroni corrections	Friedman's statistic = 17.2<br>Degrees of freedom for age = 2	p < 0.001 (main effect of age) **<br>p = 1.00 (P5 vs. P10)<br>p = 0.001 (P5 vs. P15) **<br>p < 0.001 (P10 vs. P15) **	P20 excluded
Fig. S2D	Median USV slope x age *N = 13 mice*	Friedman, post-hoc Wilcoxon-signed-rank tests with Bonferroni corrections	Friedman's statistic = 6.62<br>Degrees of freedom for age = 2	p = 0.04 (main effect of age) *<br>p = 0.57 (P5 vs. P10)<br>p = 0.051 (P5 vs. P15)	P20 excluded

				p = 0.051 (P10 vs. P15)	
Fig. S2E	Median USV spectral purity x age *N = 13 mice*	Friedman, post-hoc Wilcoxon-signed-rank tests with Bonferroni corrections	Friedman's statistic = 17.1<br>Degrees of freedom for age = 2	p < 0.001 (main effect of age) **<br>p = 0.004 (P5 vs. P10) **<br>p = 0.001 (P5 vs. P15) **<br>p = 0.001 (P10 vs. P15) **	P20 excluded
Fig. S6A (left); also reported in Fig. 4B, left	Median USV duration x non-vocal behavior at P5 *N = 11 P5 mice*	Friedman, post-hoc Wilcoxon-signed-rank tests with Bonferroni corrections	Friedman's statistic = 3.45<br>Degrees of freedom for behavior = 2	p = 0.18 (main effect of non-vocal behavior)	
Fig. S6A (middle); also reported in Fig. 4B, middle	Median USV duration x non-vocal behavior at P10 *N = 25 P10 mice*	Wilcoxon-signed-rank test	Degrees of freedom for behavior = 1	p = 0.015 (locomotion vs. lying still) *	
Fig. S6A (right); also reported in Fig. 4B, right	Median USV duration x non-vocal behavior at P15 *N = 5 P15 mice*	Friedman, post-hoc Wilcoxon-signed-rank tests with Bonferroni corrections	Friedman's statistic = 3.89<br>Degrees of freedom for behavior = 2	p = 0.14 (main effect of non-vocal behavior)	

Fig. S6B (left); also reported in Fig. 4C, left	Median USV mean pitch x non-vocal behavior at P5<br>*N = 11 P5 mice*	Friedman, post-hoc Wilcoxon-signed-rank tests with Bonferroni corrections	Friedman's statistic = 2.36<br>Degrees of freedom for behavior = 2	p = 0.31 (main effect of non-vocal behavior)	
Fig. S6B (middle); also reported in Fig. 4C, middle	Median USV mean pitch x non-vocal behavior at P10<br>*N = 25 P10 mice*	Wilcoxon-signed-rank test	Degrees of freedom for behavior = 1	p = 0.24 (locomotion vs. lying still)	
Fig. S6B (right); also reported in Fig. 4C, right	Median USV mean pitch x non-vocal behavior at P15<br>*N = 5 P15 mice*	Friedman, post-hoc Wilcoxon-signed-rank tests with Bonferroni corrections	Friedman's statistic = 0.4<br>Degrees of freedom for behavior = 2	p = 0.82 (main effect of non-vocal behavior)	
Fig. S6C (left)	Median USV FM index x non-vocal behavior at P5<br>*N = 11 P5 mice*	Friedman, post-hoc Wilcoxon-signed-rank tests with Bonferroni corrections	Friedman's statistic = 0.73<br>Degrees of freedom for behavior = 2	p = 0.695 (main effect of non-vocal behavior)	
Fig. S6C (middle)	Median USV FM index x non-vocal behavior at P10<br>*N = 25 P10 mice*	Wilcoxon-signed-rank test	Degrees of freedom for behavior = 1	p = 0.02 (locomotion vs. lying still) *	

Fig. S6C (right)	Median USV FM index x non-vocal behavior at P15 *N = 5 P15 mice*	Friedman, post-hoc Wilcoxon-signed-rank tests with Bonferroni corrections	Friedman's statistic = 4.8 Degrees of freedom for behavior = 2	p = 0.09 (main effect of non-vocal behavior)	
Fig. S6D (left)	Median mean USV frequency x non-vocal behavior at P5 *N = 11 P5 mice*	Friedman, post-hoc Wilcoxon-signed-rank tests with Bonferroni corrections	Friedman's statistic = 1.27 Degrees of freedom for behavior = 2	p =0.53 (main effect of non-vocal behavior)	
Fig. S6D (middle)	Median mean USV frequency x non-vocal behavior at *N = 25 P10 mice*	Wilcoxon-signed-rank test	Degrees of freedom for behavior = 1	p = 0.47 (locomotion vs. lying still)	
Fig. S6D (right)	Median mean USV frequency x non-vocal behavior at P15 *N = 5 P15 mice*	Friedman, post-hoc Wilcoxon-signed-rank tests with Bonferroni corrections	Friedman's statistic = 0.4 Degrees of freedom for behavior = 2	p = 0.82 (main effect of non-vocal behavior)	
Fig. S6E (left)	Median standard deviation of USV mean frequency x non-vocal behavior at P5 *N = 11 P5 mice*	Friedman, post-hoc Wilcoxon-signed-rank tests with Bonferroni corrections	Friedman's statistic = 2.36 Degrees of freedom for behavior = 2	p = 0.31 (main effect of non-vocal behavior)	

Fig. S6E (middle)	Median standard deviation of USV mean frequency x non-vocal behavior at P10<br>*N = 25 P10 mice*	Wilcoxon-signed-rank test	Degrees of freedom for behavior = 1	p = 0.02 (locomotion vs. lying still) *	
Fig. S6E (right)	Median standard deviation of USV mean frequency x non-vocal behavior at P15<br>*N = 5 P15 mice*	Friedman, post-hoc Wilcoxon-signed-rank tests with Bonferroni corrections	Friedman's statistic = 7.6<br>Degrees of freedom for behavior = 2	p = 0.02 (main effect of non-vocal behavior) *<br>p = 0.19 (locomotion vs. lying still)<br>p = 1.00 (grooming vs. lying still)<br>p = 0.19 (grooming vs. locomotion)	
Fig. S6F (left)	Median USV slope x non-vocal behavior at P5<br>*N = 11 P5 mice*	Friedman, post-hoc Wilcoxon-signed-rank tests with Bonferroni corrections	Friedman's statistic = 2.36<br>Degrees of freedom for behavior = 2	p = 0.31 (main effect of non-vocal behavior)	
Fig. S6F (middle)	Median USV slope x non-vocal behavior at P10<br>*N = 25 P10 mice*	Wilcoxon-signed-rank test	Degrees of freedom for behavior = 1	p = 0.02 (locomotion vs. lying still) *	

Fig. S6F (right)	Median USV slope x non-vocal behavior at P15 *N = 5 P15 mice*	Friedman, post-hoc Wilcoxon-signed-rank tests with Bonferroni corrections	Friedman's statistic = 0.4<br>Degrees of freedom for behavior = 2	p = 0.82 (main effect of non-vocal behavior)	
Fig. S6G (left)	Median USV spectral purity x non-vocal behavior at P5 *N = 11 P5 mice*	Friedman, post-hoc Wilcoxon-signed-rank tests with Bonferroni corrections	Friedman's statistic = 1.27<br>Degrees of freedom for behavior = 2	p = 0.53 (main effect of non-vocal behavior)	
Fig. S6G (middle)	Median USV spectral purity x non-vocal behavior at P10 *N = 25 P10 mice*	Wilcoxon-signed-rank test	Degrees of freedom for behavior = 1	p = 0.02 (locomotion vs. lying still)<br>*	
Fig. S6G (right)	Median USV spectral purity x non-vocal behavior at P15 *N = 5 P15 mice*	Friedman, post-hoc Wilcoxon-signed-rank tests with Bonferroni corrections	Friedman's statistic = 2.8<br>Degrees of freedom for behavior = 2	p = 0.25 (main effect of non-vocal behavior)	

Table S2. USV counts by age and by animal. USV counts included in Fig. 1B are presented, and mice that produced > 50 USVs in the P5, P10, and P15 recordings are indicated in bolded and italicized text (N = 13, analyzed in Fig. 1C and Fig. S2).

Mouse	P5 USVs	P10 USVs	P15 USVs	P20 USVs
1	43	351	163	0
2	91	42	15	0
3	no file	210	64	0
4	148	565	36	0
5	***164***	***149***	***157***	***0***
6	***79***	***370***	***60***	***0***
7	***296***	***574***	***51***	***11***
8	no file	403	32	0
9	***111***	***729***	***75***	***0***
10	***380***	***91***	***274***	***0***
11	***81***	***212***	***269***	***0***
12	48	585	0	0
13	41	273	18	0
14	71	209	0	0
15	96	372	0	0
16	96	335	43	6
17	38	316	96	0
18	4	392	2	0
19	363	341	8	0
20	287	447	3	0
21	***368***	***338***	***227***	***0***
22	***342***	***609***	***234***	***0***
23	282	437	30	0
24	143	390	4	0
25	65	538	37	0
26	43	198	0	0
27	***94***	***235***	***81***	***13***
28	***51***	***485***	***56***	***0***
29	***59***	***171***	***61***	***126***
30	180	169	35	0
31	***265***	***259***	***223***	***216***
32	11	no file	2	0
33	26	12	4	0
34	130	797	0	0
35	9	161	33	0
36	13	39	0	0
37	49	25	0	0

38	42	43	0	0
39	107	459	7	0
40	***138***	***69***	***293***	***0***
41	46	616	137	0
42	111	381	0	0
43	15	90	4	0
44	36	140	131	0
45	11	91	21	0
